# Two simple movement mechanisms for spatial division of labour in social insects

**DOI:** 10.1038/s41467-022-34706-7

**Published:** 2022-11-15

**Authors:** Thomas O. Richardson, Nathalie Stroeymeyt, Alessandro Crespi, Laurent Keller

**Affiliations:** 1 Department of Ecology and Evolution, University of Lausanne, Lausanne, Switzerland; 2 School of Biological Sciences, University of Bristol, Bristol, UK; 3 Biorobotics Laboratory (BioRob), Institute of Bioengineering, École Polytechnique Fédérale de Lausanne (EPFL), Lausanne, Switzerland

**Keywords:** Behavioural ecology, Ecological networks, Complexity, Emergence

## Abstract

Many animal species divide space into a patchwork of home ranges, yet there is little consensus on the mechanisms individuals use to maintain fidelity to particular locations. Theory suggests that animal movement could be based upon simple behavioural rules that use local information such as olfactory deposits, or global strategies, such as long-range biases toward landmarks. However, empirical studies have rarely attempted to distinguish between these mechanisms. Here, we perform individual tracking experiments on four species of social insects, and find that colonies consist of different groups of workers that inhabit separate but partially-overlapping spatial zones. Our trajectory analysis and simulations suggest that worker movement is consistent with two local mechanisms: one in which workers increase movement diffusivity outside their primary zone, and another in which workers modulate turning behaviour when approaching zone boundaries. Parallels with other organisms suggest that local mechanisms might represent a universal method for spatial partitioning in animal populations.

## Abstract (web summary)

Ant and honeybee workers specialize on certain tasks and also on zones within the nest; but how do they avoid straying into the wrong zone? The authors conduct automated tracking experiments following thousands of individuals, revealing that workers use context-dependent rules to navigate inside the nest.

## Introduction

Spatial heterogeneities in the distribution of organelles within cells^1^, of cells within tissues^2^, of individuals and social groups within populations^3^, and of populations within communities^4^ are ubiquitous in nature and are believed to play a fundamental role in the functioning of biological systems. A common challenge in the study of these heterogeneities is to uncover the links between global patterns and the processes operating at the level of individual units. For example, understanding how individual-level movement properties lead to the emergence of population-level spatial patterns, such as the segregation of animals into separate home ranges or territories, has been the focus of much attention in animal ecology^3,5–7^. Theoretical movement models have assumed that home ranges could be the product of animals (i) consistently biasing the direction of their movement towards the centre of their home range (*focal-point attraction*), (ii) switching between low- and high-diffusivity movement regimes depending on position (*locomotion adjustment*), or (iii) changing direction when encountering home range boundaries (*boundary effect*)^7^; other candidate mechanisms for home range behaviour include memory effects^8^, competitive interactions^9^ or conspecific avoidance^10^. Some of these mechanisms have received support from empirical studies of individual trajectories (e.g. *focal-point attraction* in desert ants^11^, *locomotion adjustment* in foraging bees^12^, nematodes^13^ and albatrosses^14^, and *boundary effect* in butterflies^15^, among others). However, the use of spatially-explicit approaches that make comparisons between empirical and simulated spatial trajectories to tease apart multiple candidate mechanisms has been a relatively recent development fraught with analytical and statistical challenges^3,5^, and it remains unclear whether the individual-level behavioural mechanisms for generating global spatial patterning are system-specific or generic^7^. Here we aim to evaluate which individual movement mechanisms may be responsible for collective patterns of spatial occupancy within the nests of four different species of social insect.

**Fig. 1:**
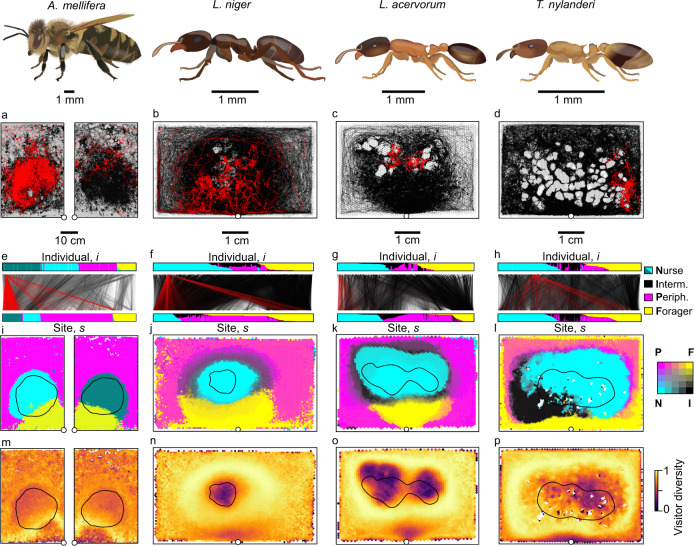
Spatial organisation in four species of social insects. **a**–**d** One-hour trajectories of all workers in an example colony. The nest interior is divided into discrete sites defined by a hexagonal grid. The trajectory of a single focal individual is shown in red. **e**–**h** Corresponding site-visit networks. Edges connect individuals (upper layer) to the sites they visit (bottom layer). Edge widths are proportional to the number of visits. The module scores for each node are indicated by the coloured bars (Nurse (N): cyan; Intermediate (I): black; Peripheral (P): magenta; Forager (F): yellow). In the honeybee colony, two different shades of cyan are used to depict the two distinct nurse modules. The connections of the focal individual are shown in red. **i**–**l** Map of the nest floor. Sites are coloured according to the linear combination of CMYK colours for each module, weighted by its module scores; thus a site with a forager score of 1 (‘pure’ forager site) is shown in yellow (top right corner in the colour key), while a site with a forager score of 0.5 and a peripheral score of 0.5 is shown in orange (middle of top row in the colour key). In the honeybee colony, two different shades of cyan are used to depict the two distinct nurse modules. Solid black lines indicate the borders of the broodnest in *A. mellifera*, and the outline the brood pile in the ants. The circle indicates the nest entrance. White grid cells correspond to unvisited sites (typically occupied by clusters of brood in the ants, or wax pillars in the bees). **m**–**p** Heatmaps showing the module score diversity of all visitors to each site (‘Visitor diversity’). The brood and the area near the nest entrance are associated with low visitor diversity (i.e., low mixing between individuals from different modules), whilst the intermediate area is associated with high visitor diversity (i.e., high mixing between individuals from different modules). White grid cells correspond to unvisited sites. Source data are provided as a source data file.

Social insects form extended-family units numbering from tens to millions of individuals, which are morphologically, physiologically and/or behaviourally differentiated into functional groups (e.g. nurses feeding and grooming brood, or foragers bringing back food to the nest) whose membership remains relatively consistent over intermediate timescales^16,17^. This social division of labour among workers is typically associated with a spatial division of the nest into task zones which are mainly visited by different subsets of workers^18–29^. Because of this almost ubiquitous relationship between social and spatial heterogeneities within the nest, social insects are an ideal system to investigate the link between individual behaviour and collective spatial patterns. Here we do so by using an automated tracking system to obtain the within-nest spatial trajectories of 12 559 individually-marked workers from colonies of the honeybee *Apis mellifera*, the common black garden ant *Lasius niger*, the large acorn ant *Leptothorax acervorum*, and the acorn ant *Temnothorax nylanderi* (Fig. 1, Fig. S1, Supplementary Note 1). To objectively describe the patterns of spatial occupancy of individuals within the nest, we develop a spatially-explicit analytical framework in which individuals and the locations they visit are represented as a two-layer (i.e., bipartite) network^30^. As this framework quantifies ties between individuals and locations (rather than between individuals and tasks^25,26,31–33^, or among individuals^17,22,28,34,35^), it combines the social and spatial structure of the colony within a single representation, providing an objective method for functional mapping of the nest into spatial ‘modules’. We then use these spatial maps and the over two billion trajectory coordinates produced by the tracking system to explore whether our empirical data are consistent with modified versions of the three main candidate mechanisms presented above. In the context of social insect nests, the *focal-point attraction* mechanism is formulated as a global navigation process in which workers consistently bias the direction of their movement towards the boundaries of their primary module when they stray outside of it. Such a global navigation process, which assumes that workers always know in what direction their goal is regardless of their current position (e.g., through landmark navigation or path integration^11,36^), has been used as a means of producing spatial organisation in models of social insect colonies^37^, but has not been experimentally demonstrated inside the nest. The *locomotion adjustment* mechanism is formulated as a context-dependent process in which workers use local cues such as chemical blends deposited onto the nest surfaces^38^ to assess whether or not they are currently within their primary module, and accordingly adopt either slow- or fast-diffusing movement. Slow-diffusing movement within the primary module would reduce workers’ likelihood of leaving it, whilst fast-diffusing movement when outside the primary module would increase their likelihood of quickly finding it. Finally, the *boundary effect* mechanism is formulated as a second context-dependent process in which workers can detect local module boundaries and actively adjust their movement direction to avoid leaving their preferred module (e.g., by making a U-turn when encountering the boundary from the inside), or to preferentially cross into it (e.g., by keeping walking straight when encountering the boundary from the outside).

Our results do not provide support for the *focal-point attraction* mechanism as a generic organisation rule, as ants do not display consistent directional bias towards their primary module and bees do so only weakly. By contrast, empirical trajectories in all four species are consistent with both the *locomotion adjustment* and *boundary effect* mechanisms, suggesting that the spatial organisation of social insect nests may be underpinned by these two well-conserved local movement mechanisms.

## Results

### Quantifying the social and spatial organisation of social insect nests

The automated video tracking produced a dataset consisting of ~ 2.5 × 10^9^ trajectory coordinates, corresponding to ~ 2.1 × 10^5^ ant-hours and ~ 1.3 × 10^5^ bee-hours of observations (Table 1).

**Table 1:** Summary statistics for colony sizes and tracking data

Species	*N* colonies	Colony size	Colony size	Total tracked	Sum total	Sum total
		(mean ± S.D.)	(range)	Individuals	Trajectory coordinates	Ant/bee hours
*A. mellifera*	10	4494 ± 1176	2867–6462	8850	970,538,883	134,797
*L. niger*	20	93 ± 35	31–139	1867	839,179,656	116,553
*L. acervorum*	10	64 ± 21	22–94	635	210,372,480	29,218
*T. nylanderi*	10	114 ± 37	76–191	1142	441,439,804	61,311

To analyse these data, the trajectories of all individually-marked workers in each colony were combined into a bipartite spatial network consisting of two types of nodes: ‘individuals’ (workers) and spatial ‘sites’ obtained by dividing the nest into a regular grid (Fig. 1a–d, Fig. S2, Supplementary Note 2). These two types of nodes were connected by weighted edges representing the number of times each individual visited each site (Fig. 1e–h). This representation thus allows the spatial habits of all colony members to be summarized in a single network.

To test whether individuals can be classified into groups with similar space use patterns, we used a stochastic community detection algorithm to partition the network into distinct modules, each consisting of a set of workers with strong ties to a shared set of sites^39^. This stochastic algorithm was repeated 1000 times for each colony to evaluate the variation in both the number of modules identified and the assignment of individuals and sites to particular modules. In all four species, colonies typically consisted of four modules (mean ± standard error of the proportion of algorithm iterations that identified exactly four modules, *A. mellifera*: 89.5 ± 0.03%; *L. niger*: 71.3 ± 0.05%; *L. acervorum*: 79.8 ± 0.04%; *T. nylanderi*: 74.4 ± 0.07%; Fig. S3). In the ants there was typically one module whose outline closely followed the border of the brood pile (‘nurse’ module), one module centred on the nest entrance (‘forager’ module), one that closely followed the internal walls (‘peripheral’ module), and one that formed a sickle- or ring-shaped area between the nurse and forager modules or between the nurse and peripheral modules (‘intermediate’ module, Fig. 1 j–l, Fig. S4-S5, Supplementary Note 3). The honeybee colonies also typically consisted of four modules, including one peripheral and one forager module, but instead of one nurse module and one intermediate module, they possessed two nurse modules – one covering the broodnest on one side of the wax comb, and the other covering the broodnest on the other side of the comb (Fig. 1 i, Fig. S5). The presence of two distinct nurse modules in the honeybee probably stemmed from the physical architecture of the honeybee nest (a double-sided wax comb with a separate patch of brood cells on each side^20^), combined with the relatively low mobility of nurse bees, which together limit the exchange of nurses between the two broodnests (mean ± standard error of the number of switches between the two sides of the comb, nurses: 15.2 ± 0.26; other bees: 35.6 ± 0.51; generalized linear mixed-effects model (GLMM) with Poisson error & colony identity as random effect, nurses versus all other bees: d.f. = 1, *χ*^2^=33721, *p* < 0.0001). However, bees from the two nurse modules did not differ from one another in age (general linear mixed-effect model (LME) with mean module age as a main effect and & colony identity as random effect, difference between the two nurse modules: *z* = 0.93, *p* = 0.79). Therefore, in all subsequent analyses the two honeybee nurse modules were pooled into a single group.

Repeated iterations of the community detection algorithm revealed that individuals and sites were often allocated to more than one module. To quantify this overlap between modules, each individual and each site was assigned four continuous ‘module scores’, representing the proportion of iterations in which the node was assigned to each module (Fig. 1 e–l, Fig. S2, Fig. S4-S5). As these measures are proportions, module scores vary in the range 0-1, and the four scores of each individual or site always sum to 1. Thus, an ant that was most often assigned to the forager module and occasionally to the peripheral module, would have a high score for the forager module (its ‘primary’ module, i.e., the module for which it scores highest), a low score for the peripheral module, and zero scores for the nurse and intermediate modules. In all four species, the majority of individuals had either the nurse or the forager module as their primary module, with the intermediate and peripheral modules making up the remaining 20-30% of the individuals, and the remaining 30-40% of the sites (Table S1, Supplementary Note 4).

**Fig. 2:**
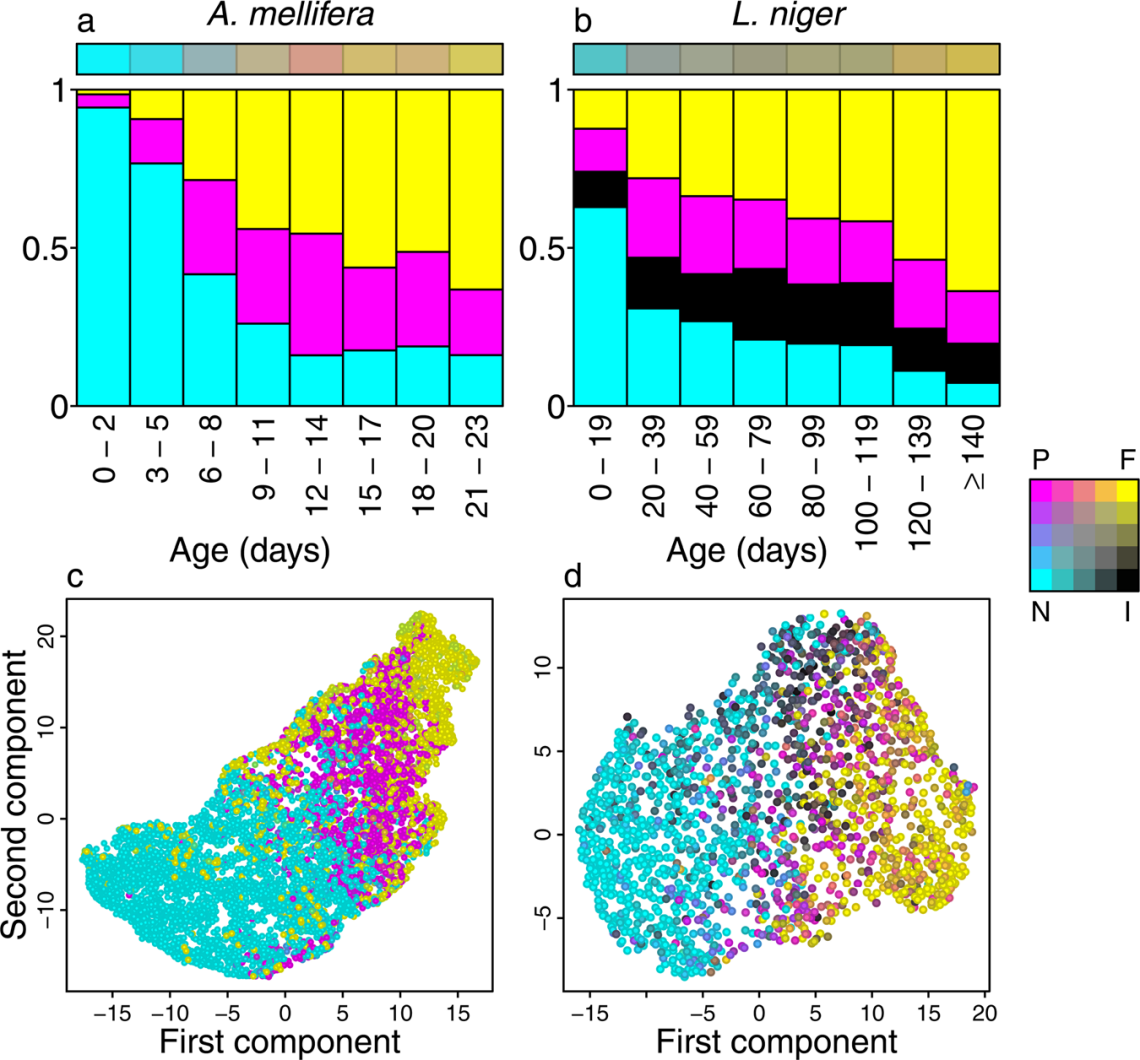
Correlations between age, module score and task validate the spatial approach. **a**, **b** Honeybee and ant workers exhibit similar transitions between modules as a function of age. Stacked bars indicate the average module score profile for individuals of a given age. The horizontal bar encodes the typical module score profile for individual of each age (CMYK combination of the scores for each module, averaged across all individuals of each age). **c**, **d** Workers belonging to different spatial modules exhibit different task profiles. Scatterplots represent the outcome of LargeVis dimensionality reductions applied to the worker task profiles. Points represent different individuals. Point separation indicates task profile similarity. The module scores were not used in the LargeVis analysis or in the task definitions. See Supplementary Movie 1 for three-dimensional LargeVis representations of all four species. Source data are provided as a source data file.

To assess the performance of our community detection approach we performed two validation steps. The first step was to check for a correlation between age and task (i.e., age polyethism), which is observed in most species of social insects^18–20^. Both species for which we had data on individual age (i.e., *A. mellifera* and *L. niger*) exhibited characteristic signs of age polyethism: young workers had the highest scores for the nurse module, middle-age workers had the highest scores for the peripheral and intermediate modules, whilst old workers had the highest scores for the forager module (Fig. 2a–b).

As social insect division of labour is characterised by different worker groups carrying out different sets of tasks in different parts of the nest^18,20,28^, our second validation step was to check whether the spatial module(s) to which an individual belongs correspond to the tasks it performs, that is, its ‘task profile’. To quantify worker task profiles we analysed several task-relevant behaviours: patrolling, queen attending, foraging, and entrance guarding. Of these, patrolling, queen attending and foraging were defined without any reference to workers’ position within the nest, whilst entrance guarding was defined based on workers’ behavioural state when just inside the nest entrance (see Methods). To visualise the individual-level variation in these high-dimensional task profiles we used the LargeVis algorithm^40^ to reduce the task profiles to a two-dimensional ‘task space’. In this representation, each point represents a worker, and the distance between pairs points represents the dissimilarity in their task profiles. Hence, workers with similar task profiles are close to one another, and workers with dissimilar task profiles are far apart. In all four species individuals with similar module scores clustered together in the task space (Fig. 2c–d; Supplementary Movie 1). In all species, the nurses and foragers each clustered together on opposite sides of the task space, suggesting that they had the most dissimilar task profiles. The intermediates and peripherals clustered together in the area between the nurses and foragers, indicating that their task profiles were a combination of those exhibited by the nurses and the foragers. Multiple analysis of variance (MANOVA) confirmed that individuals with different primary modules had significantly different task profiles (Fig. S6, Supplementary Note 5), and trajectory analysis further indicated that they displayed significantly different movement characteristics (Fig. S7, Supplementary Note 6), confirming that the modules identified using our purely spatial approach correspond to biologically-relevant functional groups.

### Comparative analysis of social and spatial organisation

**Fig. 3:**
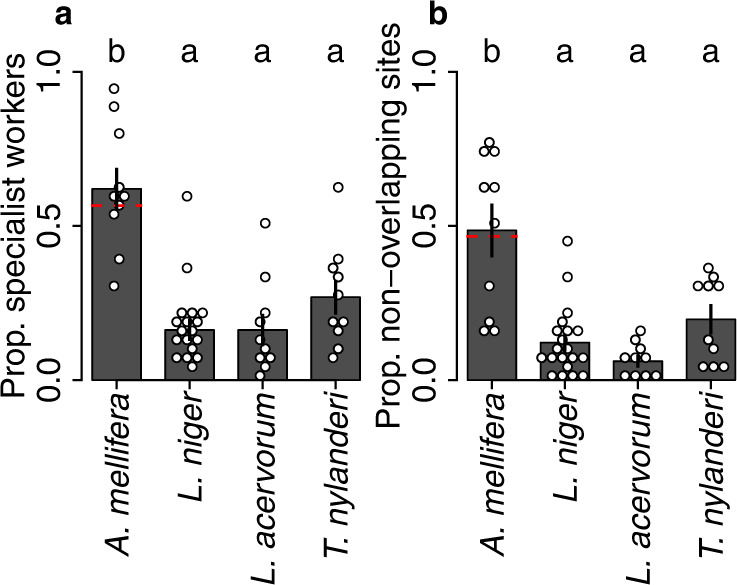
Honeybee colonies exhibit greater socio-spatial segregation than ant colonies. Points represent colony means. Bars & whiskers indicate grand means and standard errors. **a** Proportion of specialist workers (individuals that belong to only one module). The red line shows the case when the two *A. mellifera* nurse modules were treated separately, rather than being pooled. General linear model (GLM); proportion of specialist workers ~ species, *F*_3,46 _= 15.7, *p* < 0.0001. Letters above bars indicate Benjamini-Hochberg (BH) corrected post-hoc contrasts between all species pairs. Three ant versus honeybee contrasts: ∣*z*∣ > 3.8, *p* < 0.001 in each case. **b** Proportion of non-overlapping sites (sites that belong to only one module). GLM; proportion of non-overlapping sites ~ species, *F*_3,46 _= 16.8, *p* < 0.0001. Three ant versus honeybee contrasts: ∣*z*∣ > 4.3, *p* < 0.0005 in each case. All analyses based on *n* = 50 biologically independent colonies. Source data are provided as a source data file.

Examining the module scores of all sites and individuals revealed that honeybee colonies exhibited greater social and spatial segregation than ant colonies. Indeed, compared to ants, honeybee colonies had a higher proportion of full specialists (i.e., workers belonging to only one module, Fig. 3 a, Fig. S2; Table S2, Supplementary Note 7). Similarly, honeybee colonies had a larger proportion of non-overlapping sites (i.e., sites belonging to only one module) than ant colonies (Fig. 3 b, Fig. 1 i–l, Fig. S2, Fig. S4; Table S2). The greater social and spatial segregation of honeybee colonies was not contingent upon the pooling of the two honeybee nurse modules (Fig. 3, red lines; Table S3), nor was it a methodological artefact arising from differences in network sizes between species (Fig. S8, Supplementary Note 8). Though this greater social and spatial segregation in the honeybees could be due to their larger colony sizes leading to more pronounced division of labour^41–43^, it was not possible to tease apart the relative effect of colony size versus species idiosyncrasies in our dataset (Supplementary Note 7).

**Fig. 4:**
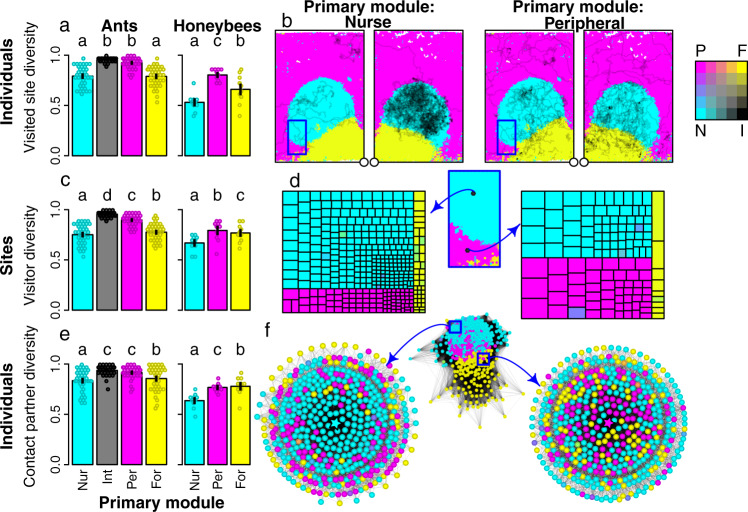
Quantifying the diversity of visited sites, site visitors, and contacted nestmates. Barplots show the module score diversity of the sites an individual visited, D_*visited*,*i*_ (**a**), of the visitors to a given site, D_*visitor*,*s*_ (**c**), and of the nestmates an individual physically contacted, D_*contacted*,*i*_ (**e**). Diversity was quantified as an entropic measure of heterogeneity, ranging from 0 (all visited sites, visitors, or contact partners belong to the same module) to 1 (even representation of visited sites, visitors, or partners from all modules). Points represent colony means. Bars *&* whiskers indicate the grand means and standard errors. Letters above bars indicate BH corrected post-hoc contrasts, derived from linear mixed-effect models (LME) predicting diversity by module. Colony identity was used as a random effect in all models. Models were fitted separately for the ants and the bees because they differed in the identity of their modules. D_*visited*,*i*_ ~ module, honeybees: *χ*^2^ = 1764.0, d.f. = 2, *p* < 0.0001; ants: *χ*^2^ = 895.8, d.f. = 3, *p* < 0.0001; post-hoc contrasts, ∣*z*∣ ≥ 18.8, *p* < 0.0001 in all cases. D_*visitor*,*s*_ ~ module, honeybees: *χ*^2^ = 30121, d.f. = 2, *p* < 0.0001; ants: *χ*^2^ = 58279, d.f. = 3, *p* < 0.0001; post-hoc contrasts, ∣*z*∣ ≥ 13.2, *p* < 0.0001 in all cases. D_*contacted*,*i*_ ~ module, honeybees: *χ*^2^ = 3497.2, d.f. = 2, *p* < 0.0001; ants: *χ*^2^ = 680.2, d.f. = 3, *p* < 0.0001; post-hoc contrasts, ∣*z*∣≥8.4, *p* < 0.0001 in all cases. **b** Three-day trajectories for an example nurse (left) and peripheral (right) honeybee workers on both sides of the wax comb. The circles indicate the nest entrance. **d** Worker visitation patterns for the two example focal sites indicated in the module map blow-up (left: nurse site; right: peripheral site). Visitation patterns represented as treemaps; each rectangle represents a worker that visited the focal site at least once. Rectangle areas are proportional to the number of visits by a worker to the site. Colours encode each worker’s module scores (CMYK combination). Rectangles are grouped together according to workers' primary module. **f** The middle network shows the daily contact network, in which nodes represent individuals, and weighted edges the number of physical contacts between bees. Node positions are determined by a force-directed layout. The left and right plots show ego-centric contact networks for a nurse (left) and a peripheral bee (right), indicated by the central stars. The distance from each node to the focal bee indicates the number of contacts with the focal bee. Source data are provided as a source data file.

To quantify the mixing between worker populations from different modules, we calculated an entropy-based measure of heterogeneity, termed ‘module score diversity’ (see Methods), for (i) all the sites that each individual visited, (ii) all the individuals that visited a given site (Fig. 1 m–p, Fig. S9, Supplementary Note 9), and (iii) all the nestmates that each individual physically contacted. Workers from the peripheral or intermediate modules visited a greater diversity of sites than those from the nurse or forager modules (Fig. 4a–b; Table S4, and Supplementary Note 10). Similarly, sites from the intermediate or peripheral modules were visited by a greater diversity of individuals than sites from the nurse or forager modules (Fig. 4 c–d; Table S4). Finally, workers from the intermediate or peripheral modules interacted with a greater diversity of nestmates than workers from the nurse or forager modules (Fig. 4e–f; Table S4). Overall, these results indicate that the intermediate and peripheral modules may play an ‘interface’ role in mediating mixing between different worker populations.

Finally, as the brood and the nest entrance are key locations within the nest, we tested whether these areas were subject to more or less mixing than other areas. Across all species both areas had a lower diversity of visitors than other parts of the nest (Fig. 1 m–p; Fig. S9; LME with visitor diversity as response, colony as random effect, and proximity to brood, nest entrance and colony size as main effects; effect of brood: *β* ≤ −0.17, *χ*^2^ ≥ 4829.2, *p* < 0.0001; effect of nest entrance: *β* ≤ −0.17, *χ*^2^ ≥ 1354.3, *p* < 0.0001 in all species; Table S5). Thus, the brood and entrance areas appear to be exclusive zones that are visited primarily by specialist workers from a single module (the nurse and forager modules respectively), whereas other nest areas are mixing zones that are visited by generalists and/or specialists from multiple modules.

### Describing the movement patterns associated with collective spatial organisation

To explore how nest-level spatial segregation may arise from individual movement patterns, we tested predictions from the three candidate movement mechanisms described above: *focal-point attraction*, *locomotion adjustment* and *boundary effect*. The *focal-point attraction* mechanism predicts that individuals should bias the direction of their movement towards their primary module over both short and long distances. The *locomotion adjustment* mechanism predicts that fundamental statistics that underpin locomotion diffusivity (probability of being active, speed and turn angle) should vary depending upon location, and whether individuals are inside or outside their primary module. The *boundary effect* mechanism predicts that when reaching the boundary of their primary module, individuals should vary their turning behaviour depending upon their approach direction, such that they make larger (respectively smaller) turns when approaching the boundary from the inside (respectively outside).

**Fig. 5:**
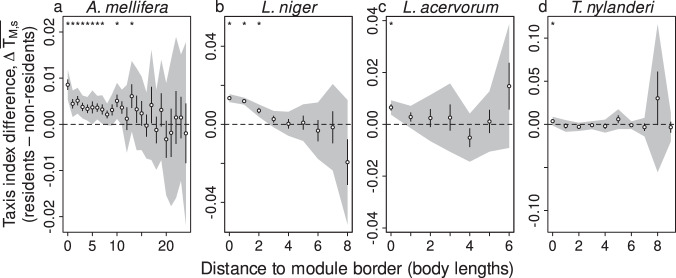
Quantifying directional bias toward the primary module. Panels **a**–**d** show the signed difference in the taxis indices of resident versus non-resident workers as a function of distance to the focal module border, for each species. Positive values occur when individuals from a focal module (residents) exhibit stronger taxis toward the nearest point on the border of that module than individuals from other modules (non-residents). Vertical bars indicate standard errors, and shaded areas represent the 95% confidence intervals after Bonferroni correction. Asterisks indicate distances at which there was a statistically significant difference between the taxis of residents and non-residents after BH correction for multiple testing. All analyses based on *n* = 12494 workers, drawn from *n* = 50 colonies. Source data are provided as a source data file.

First, to assess whether empirical trajectories are consistent with the *focal-point attraction* mechanism, we tested whether the directional bias (‘taxis index’; see Methods, Fig. S10-S11 and Supplementary Note 11) towards any focal module was greater in workers belonging to that module than in other workers. Honeybees exhibited some directional bias towards their primary module up to distances of 13 body lengths outside the boundary; however, this effect was very weak and completely absent at longer distances (Fig. 5a). Furthermore, in all three ant species, workers only showed bias towards their primary spatial module when very close to the border (i.e., no more than 2 body lengths outside; Fig. 5 b–d). Overall, these data indicate that long-range attraction toward the primary module is very weak in honeybees and absent in ants, suggesting that *focal-point attraction* is unlikely to be a general organising mechanism for spatial organisation in social insect nests.

**Fig. 6:**
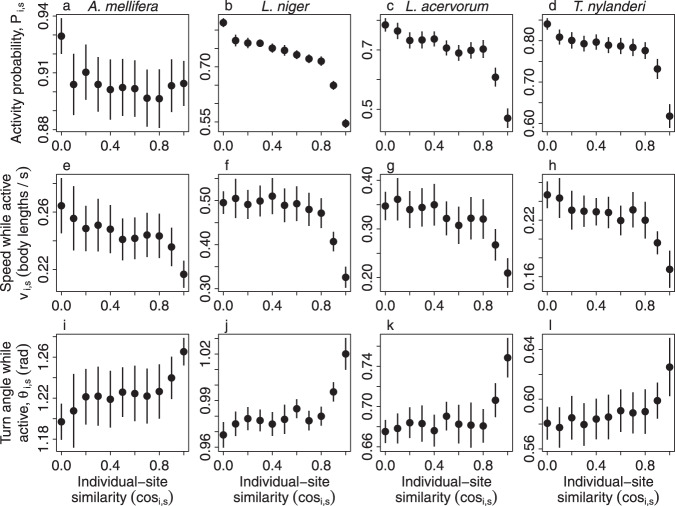
Individuals increase their diffusivity when outside their primary module. **a**–**d** Individuals were less active when visiting sites with module scores that were similar to their own, and more active when visiting sites with module scores that were very different to their own. **e**–**l** Active individuals moved more slowly and tortuously when visiting sites with module scores that matched their own, and faster and straighter when visiting sites with different module scores. Points *&* error bars represent means and standard errors, calculated across all colonies. Source data are provided as a source data file.

**Fig. 7:**
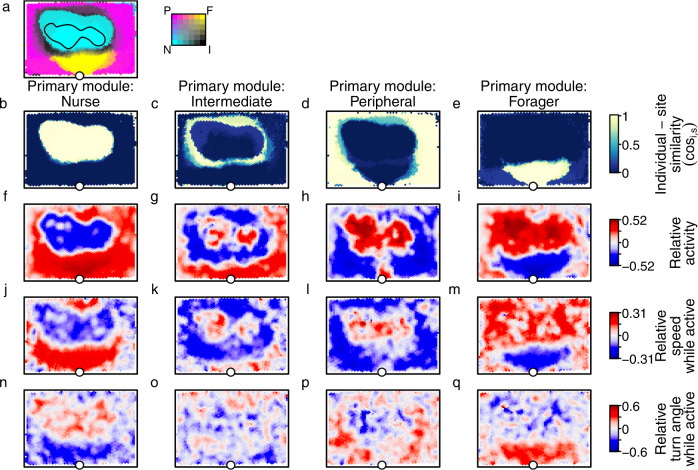
Visualising location-dependent *locomotion adjustment*. All panels show data for a representative *L. acervorum* colony. **a** Map showing the partitioning of the nest into four spatial modules. **b**–**e** Maps depicting the individual-site similarity between the observed module scores of all sites and those of a visiting worker that scored 1 for the indicated module. **f**–**q** Maps depicting spatial variation in movement parameters according to primary module. Site colours show movement metrics for individuals with the indicated primary module. To facilitate comparisons between modules, all three movement metrics are expressed as relative measures, i.e., signed differences between the mean activity probability (**f**–**i**), mean speed (**j**–**m**), or mean turn angle (**n**–**q**) calculated across all visits by individuals with the indicated primary module, and the global mean calculated across all visits by all individuals. Positive values (red) indicate a higher activity probability, a higher speed, or a greater turn angle than the global average. Workers exhibit low diffusivity (relatively low activity probabilities, slow and tortuous movement) when visiting sites with similar module scores to their own (for example, nurse workers inside the brood pile (**f**, **j**, **n**) or foragers near the nest entrance (**i**, **m**, **q**)). By contrast, workers exhibit high diffusivity (relatively high activity probabilities, fast and straight movement) when visiting site with module scores that do not match their own (for example, nurse workers near the nest entrance (**f**, **j**, **n**) or foragers inside the brood pile (**i**, **m**, **q**)). Source data are provided as a source data file.

We next tested whether the probability of a worker being active and its speed and turn angle while active depended upon location, as predicted by the *locomotion adjustment* mechanism. To account for the overlapping nature of the spatial modules, we defined the ‘individual-site similarity’ (cosine similarity between the module scores of a worker and a site, see Methods), which provides a fine-grained quantification of the degree to which a worker is inside or outside its primary module. We found that when visiting sites with module scores similar to their own, workers (i) were less likely to be in the active state (Fig. 6 a–d), (ii) moved more slowly while active (Fig. 6 e–f), and (iii) made larger turns while active (Fig. 6 i–l), than when visiting sites with module scores dissimilar to their own (Fig. S12, Supplementary Note 12; LME with individual-site similarity and colony size as main effects and colony identity, worker identity, site identity & worker density as random effects; effect of individual-site similarity on probability of being active : *χ*^2^ > 51396, d.f. = 1, *p* < 0.0001 in all species; effect of individual-site similarity on speed while active: *χ*^2^ > 20421, d.f. = 1, *p* < 0.0001 in all species; effect of individual-site similarity on turn angle while active: *χ*^2^ > 8286, d.f. = 1, *p* < 0.0001 in all species). To check these results, we mapped how these three locomotion parameters vary in space, for individuals with different primary modules (Fig. 7). These module-specific maps confirmed that workers from all four modules displayed lower diffusivity (more inactive, slower, with sharper turns) when visiting sites belonging to their primary module (where the individual-site similarity was high), and higher diffusivity (more active, faster, with smaller turns) when visiting sites outside their primary module (where the individual-site similarity was low). This transition from a low-diffusivity movement regime in the core of the primary module to a high-diffusivity movement regime when far outside it was observed in all four species and is consistent with the *locomotion adjustment* mechanism. This suggests that *locomotion adjustment* mechanism could potentially act as a general organising rule underlying spatial organisation in social insect nests.

**Fig. 8:**
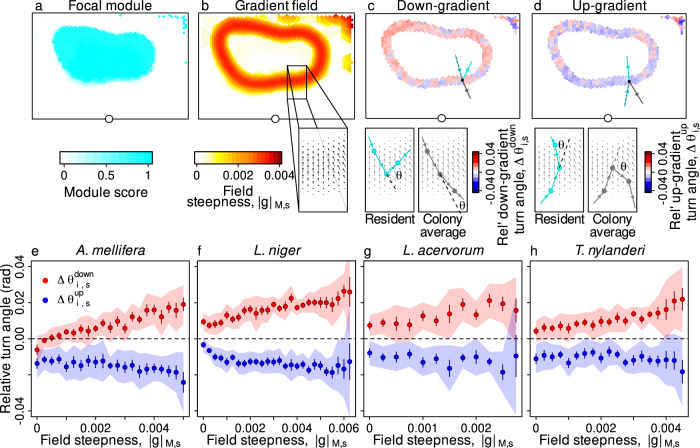
Worker navigation within module gradient fields. Panels (**a**–**d**) illustrate the direction-dependent modulation of turning behaviour within the gradient field for an example focal module (the nurse module of the same *L. acervorum* colony as shown in Fig. 7). **a**, **b** Calculating the gradient field. **a** Module score map for the focal module. **b** The focal module gradient field, derived from local spatial regressions on the site scores. The blow-up shows a transect across the border. Arrow heading and length indicate respectively the field direction and steepness. **c**, **d** Relative turn angles for residents (in this example, nurses) and non-residents heading down- and up-gradient in the field of the focal (nurse) module. Blow-ups in (**c**): residents heading down-gradient in the field of their primary module (${\bar{\theta }}_{i,s,{M}_{i}}^{down}$) exhibit larger turn angles than the average worker heading down-gradient at the same site in the same field (${\bar{\theta }}_{s,{M}_{i}}^{down}$), and so generally have positive relative turn angles (i.e., are less likely than average to leave the focal module). Blow-ups in **d**: residents heading up-gradient (${\bar{\theta }}_{i,s,{M}_{i}}^{up}$) exhibit smaller turn angles than the average worker (${\bar{\theta }}_{s,{M}_{i}}^{up}$) and so generally have negative relative turn angles (i.e, are more likely to enter the focal module). **e**–**h** Relative turn angles for workers heading down- or up-gradient (red & blue respectively) of their primary module, as a function of the gradient field steepness. All modules and all workers were included in this analysis. Points & error bars represent grand means and standard errors, calculated from the 50 colony means. Shaded areas represent the 95% confidence intervals after Bonferroni corrections. The dashed line represents the null expectation that residents and non-residents behave in the same way when approaching the module border. All analyses based on *n* = 613100 sites, and *n* = 12494 workers, drawn from *n* = 50 colonies. Source data are provided as a source data file.

Finally, we tested whether, upon reaching the boundary of their primary module, workers vary their turning behaviour depending upon their approach direction, as predicted by the *boundary effect* mechanism. To do so, we first developed a method to objectively quantify approach direction near fuzzy, overlapping module boundaries. In this method, the topography of the score landscape of each module *M* is described using a gradient field ${\overrightarrow{{{{{{{{\rm{F}}}}}}}}}}_{M}$, in which vectors encode the local score gradient steepness and direction at each site. For example, when module *M* exhibits a very gradual spatial transition into another module, sites in this transition zone are associated with short vectors (shallow gradient) pointing ‘up’ the local module score gradient (Fig. 8a–b; see Methods). We used these gradient fields to quantify how workers change direction in areas of transition between modules (i.e., areas with non-zero gradient) depending on their approach direction. In all four species, workers had higher turn angles when travelling down-gradient of their primary module compared to workers from other modules travelling in the same direction at the same location (Fig. 8c, e-h). In other words, workers approaching their primary module boundary from the inside had a greater tendency to change direction to avoid crossing the boundary and thus avoid leaving their primary module. Furthermore, in all four species workers had lower turn angles when travelling up-gradient of their primary module than workers from other modules travelling in the same direction at the same location (Fig. 8c, e-h). In other words, workers approaching their primary module boundary from the outside had a greater tendency to maintain their movement direction to cross the boundary and re-enter their primary module (Fig. 8d, e-h). Interestingly, these effects were more pronounced when the local module score gradient was steeper (Fig. 8e–h; LME on ${\bar{\theta }}_{i,s,{M}_{i}}$ with three main effects: up/down-gradient approach direction, local field steepness ∣*g*∣_*M*,*s*_, colony size, with one interaction: direction × ∣*g*∣_*M*,*s*_, and with four random effects: colony identity, worker identity, site identity and worker density; effect of direction × ∣*g*∣_*M*,*s*_: *χ*^2^ > 146.6, d.f. = 1, *p* < 0.0001 in all species). These results show that the empirical trajectories are consistent with a *boundary effect* in all four species, and that this effect is enhanced when workers encounter a discrete and well-delineated border, compared to workers that find themselves in a gradual transition between overlapping modules. This suggests that the *boundary effect* mechanism could also potentially act as a general organising rule underlying spatial organisation in social insect nests.

### From movement patterns to spatial segregation: agent-based modelling

**Fig. 9:**
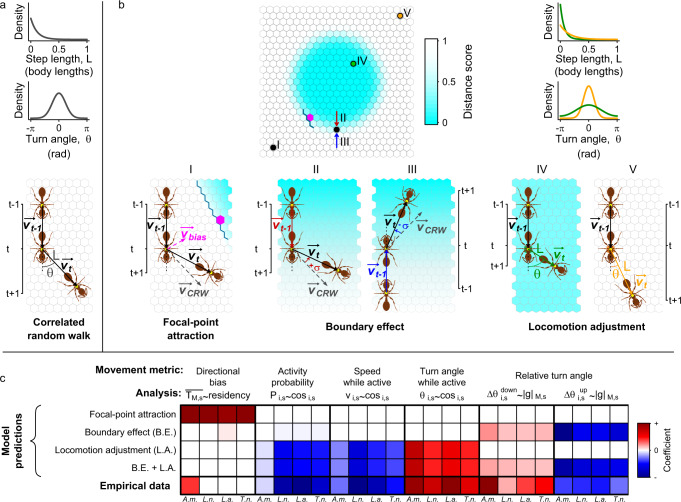
Agent-based modelling of individual movement. **a** All simulation trajectories were based on the correlated random walk (CRW) model. A CRW trajectory is constructed from a sequence of vectors $\overrightarrow{{v}_{t}}$, produced by randomly drawing a step length *L* and a turn angle *θ* from an exponential and a wrapped normal distribution, respectively. **b** The upper map represents the nest grid upon which trajectories are simulated. The nest includes two overlapping ‘spatial modules’ (central and peripheral), characterised by a ‘distance score’ increasing non-linearly with the distance of each site to the nest centre. To model the three movement mechanisms, the CRW is modified as follows. In the focal-point attraction model (point I in map), the CRW vector $\overrightarrow{{v}_{CRW}}$ is modified by adding a bias vector $\overrightarrow{{v}_{bias}}$, which points towards the closest point on the border of the individual’s primary module (magenta cell). In the *boundary effect* model (points II–III), the CRW is modified in the vicinity of the module boundary by increasing (decreasing) the CRW turn angle by *σ* when approching the boundary from the inside (outside). In the *locomotion adjustment* model, the *L* and *θ* distributions are modified according to location, so individuals make shorter (larger) steps and larger (smaller) turns when inside (outside) their primary module (points IV *&* V, green *&* orange distributions, respectively). **c** Comparison between empirical and simulated trajectories. Grouped columns indicate the five movement metrics described in the main text, and letters below individual columns give species name abbreviations. Formulas provide a brief description of the statistical model conducted for each movement metric, and cell colours encode the sign and value of the coefficient of the predictor in the statistical model (see text for the model definitions). To facilitate comparisons within metrics, the coefficients are normalised by the maximum absolute value of all coefficients for each metric. Source data are provided as a source data file.

We next developed an agent-based simulation model for individual movement, to (i) assess whether the three candidate mechanisms make distinct predictions for individual movement, and (ii) test whether the two local movement mechanisms *locomotion adjustment* and *boundary effect* are sufficient to explain the observed spatial segregation between task groups (see Supplementary Note 13 for detail). All simulations were based on a simple correlated random walk model^37^, which was modified to include one or more of the three movement mechanisms presented above, and parameterised using tracking data from each species (Table S6). Figure 9a, b illustrates the concepts underlying our modelling approach for each of the three mechanisms.

Comparing the simulation outcomes from the three candidate mechanisms confirmed that each mechanism makes distinct predictions for individual movement. Thus, only the *focal-point attraction* mechanism predicted long-range attraction toward the primary module, only the *locomotion adjustment* mechanism predicted location-dependent changes in movement diffusivity, and only the *boundary effect* mechanism predicted that turning behaviour of workers approaching their primary module boundary depends upon the approach direction and transition steepness (Figs. 9c and S13–S15). These non-overlapping predictions demonstrate that the three candidate movement mechanisms are non-redundant, so for example, the *locomotion adjustment* mechanism does not produce an apparent *boundary effect*.

Comparisons between the empirical and simulated trajectories (Fig. 9c) showed that the global *focal-point attraction* model failed to reproduce the universal movement patterns highlighted in our analyses of empirical trajectories, confirming that this mechanism is unlikely to be the main driver of spatial organisation in social insect nests. By contrast, the empirical trajectories were more consistent with simulated trajectories produced from the local mechanisms: the *locomotion adjustment* model successfully reproduced the location-dependent modulation of movement diffusivity, and the *boundary effect* model successfully reproduced the direction-dependent modulation of turning behaviour for individuals at the boundary of their primary module. However, the best match between the empirical and simulated trajectories was achieved by a model that combined both local mechanisms (B.E. + L.A. in Fig. 9c and Figs. S13–S15).

Furthermore, analyses of the spatial segregation produced by the movement models showed that the combination of the two local mechanisms is also sufficient to produce full spatial segregation between populations of workers in all species (Fig. S16). Further sensitivity analyses revealed that the *locomotion adjustment* mechanism has a stronger effect than the *boundary effect* mechanism, but both mechanisms were necessary to produce full spatial segregation in honeybee colonies (Fig. S17). Overall, our results suggest that spatial division of labour in social insect nests is consistent with workers basing their movement decisions upon two well-conserved context-dependent movement mechanisms.

## Discussion

A common feature of many social insect species is a spatial division of labour, whereby worker subsets carrying out different task sets occupy different parts of the nest^20–24,28,29,44^. The bipartite spatial network approach presented here provides an objective method for simultaneously dividing the colony population into social groups and mapping the nest into functional zones, opening the way to new quantitative analyses of socio-spatial organisation in animal groups. Although our method used spatial visitation patterns as the only input, the social groups identified differed not only in their typical location, but also in age, in the tasks they performed and in the way they moved. The connection between these different measures suggests that our approach is successful in identifying biologically-relevant social groups using trajectory fixes only, without relying on ad-hoc definitions for what constitutes a task^20,21,25,28,33,45^, a social interaction^22,23,26,28,29,44^, or a caste^23,50^. Because of its generality, and because it requires few definitions or assumptions, our approach could thus allow more meaningful comparisons between the social organisation of multiple species. Remarkably, the application of our method to four physically monomorphic species of social insect consistently identified four partially overlapping worker groups (Figs. 1 and S2–S5), which contrasts with the cross-species variability in social structure highlighted by previous studies (one worker group^46,47^, two^27,29^, three^18,22,23,45,48–50^, four^20,21,25^, or five^51^).

Our spatial approach also revealed that all four species exhibited a similar segregation between exclusive zones (around the brood and nest entrance) that were chiefly visited by single-module specialists, and interface zones (peripheral and intermediate modules) visited by generalists and/or specialists from all modules, where workers mixed with a wide range of individuals from all modules. Furthermore, the movement of nurses and foragers was more constrained to their primary module than the movement of peripheral and intermediate workers which roamed more freely across spatial modules. The tendency of nurses and foragers to restrict their movements to separate exclusive zones could have implications for disease transmission^24,52^, as it should decrease the frequency of direct contacts between high-value individuals (i.e., the queen, brood and young nurses) and high-risk individuals (i.e., foragers that may pick up pathogens outside). Although the exact function of the intermediate and peripheral individuals remains unclear, their free-roaming movement suggests that they have the opportunity to act as an interface that mediates or regulates the exchange of information and material between the otherwise less-well connected forager and nurse module.

We investigated three alternative, non-exclusive candidate behavioural mechanisms for the maintenance of spatial segregation between groups of workers: (i) a *focal-point attraction* mechanism in which workers know the direction of their primary module relative to their current location and can bias their movement towards it^36^, (ii) a *locomotion adjustment* mechanism in which individuals can adjust the diffusivity of their movement depending on whether or not they are within their primary module, and (iii) a *boundary effect* mechanism in which individuals can detect module borders and actively avoid leaving, or preferentially enter, their primary module. Our analysis provided only limited evidence that the *focal-point attraction* mechanism is used by honeybee workers, and no evidence for its use in the three ant species. By contrast, the empirical data were consistent with the *locomotion adjustment* and the *boundary effect* mechanisms in all four species, and agent-based modelling confirmed that the combination of these two mechanisms should be sufficient to maintain colony-level spatial segregation in all four species.

In the *locomotion adjustment* mechanism, workers modulate their movement diffusivity according to their location. Importantly, this simple mechanism does not require complex navigational capabilities such as path integration^11^ or cognitive maps^53^. Instead, it would be sufficient for an individual to sense local cues indicating whether or not it is within its primary module, and adjust its movement accordingly. For example, individuals may use chemical signatures as locational cues: in ants, different areas within the nest are marked by distinctive cuticular hydrocarbon blends laid on the nest floor^38^, and in honeybee colonies the wax comb is delineated into separate zones containing either developing brood, pollen, or honey, each with its own unique chemical bouquet^54^. Alternatively, interactions between task stimuli and individual response thresholds^55^ may also be responsible for the location-dependent changes in movement diffusivity. For example, within the brood pile or broodnest, a nurse with a low threshold for stimuli associated with hungry brood may be more likely to stop and attend to the brood (thus adopting slow, tortuous movement), whereas a forager with a high brood care threshold would more likely ignore the brood and continue on its original path (displaying faster, straighter movement). In such a case the movement patterns described here would represent a consequence - rather than a cause - of the underlying division of labour between workers.

In the *boundary effect* mechanism, individuals modulate their turning angle depending on the direction from which they approach the boundary of their primary module. The dependency of this response upon the steepness of the boundary suggests that individuals may respond to the local gradient in chemical cues associated with their primary module rather than to a tangible, clearly delineated entity. Although the detailed underlying mechanism remains to be established, previous studies of pheromone trail following behaviour have shown that ant workers can detect lateral differences in antennal sensory inputs and adjust their movement accordingly^56,57^, showing that they possess the neural circuitry to detect chemical gradients.

The simplicity of the two local movement mechanisms (*locomotion adjustment* and *boundary effect*) indicates that they might be present in a range of biological systems. Indeed, the *locomotion adjustment* mechanism bears a striking resemblance to ‘win-stay, lose-shift’ strategies in animal decision-making^58^, exemplified by foraging nematodes^13^, bumble bees^12^, and albatrosses^14^. Furthermore, movement consistent with the *boundary effect* mechanism has also been reported in the butterfly *Proclossiana eunomia*. In this species, individuals make ‘U’-turns when they encounter the boundary of a suitable habitat patch from the inside, which causes them to stay inside the patch^15^. Finally, *Escherichia coli* bacteria exhibit movement patterns that are highly reminiscent of the two local movement mechanisms. In these bacteria, cells immersed in an amino-acid gradient tend to make long straight ‘runs’ when facing up the gradient, but perform more frequent ‘tumbles’ (shorter runs with frequent reorientations) when facing down the gradient, causing the cell to perform a biased random walk toward the resource^59,60^.

Only honeybee colonies displayed long-range attraction toward the primary module, in agreement with the *focal-point attraction* mechanism. This additional movement mechanism in the bees could explain why honeybee colonies exhibit stronger spatial segregation than all three ant species. Alternatively, more pronounced partitioning of the honeybees could have two other explanations. First, the size of the worker populations in the honeybee colonies were about two orders of magnitude larger than those of the ant colonies (Table 1). It has been suggested that larger social insect colonies should display greater division of labour and greater specialisation of workers into tasks, leading to stronger social and spatial segregation within the nest^41–43^. However, while we could not rule out that this is the case, our experimental data provided little evidence for a direct effect of colony size on segregation. Second, the spatial segregation between the honeybee worker groups may be enhanced by the physical organisation of the honeybee hive, which is divided into discrete zones in which the comb cells contain different, easily identifiable substances, such as honey, pollen and developing brood^20,23^, and which could serve as a scaffold for modulating worker movement. As ant nests lack comb cells, the changes in chemical cues are likely to be more gradual, which could explain the greater degree of overlap. Carefully-controlled experiments that manipulate colony size and cue availability will be required to distinguish between these alternative explanations.

Overall, our study reveals that two simple context-dependent movement mechanisms are sufficient to explain the maintenance of spatial segregation in colonies of four social insect species housed in simple, two-dimensional observation nests. These results do not rule out the possibility that more complex mechanisms may be at play in more realistic, three-dimensional nests composed of multiple chambers and tunnels. Nevertheless, parallels between the movement mechanisms outlined here and movement patterns reported in a diverse range of organisms, including species with limited cognitive capacities such as bacteria and nematodes^12,13,15,59,60^, suggest that navigation algorithms based on local, context-dependent movement rules that generate population-level spatial heterogeneities may be ubiquitous in Nature.

## Methods

### Automated tracking of four social insect species

Fifty queenright colonies were used in the tracking experiments (Table 1). Honeybee colonies (subspecies *A. mellifera carnica*) were housed in the campus apiary of the University of Lausanne. Colonies of *L. niger* were raised from single mated queens collected on campus. *T. nylanderi* colonies were collected from the University of Lausanne campus, and *L. acervorum* colonies collected from Anzeindaz, Switzerland. These four species were chosen because of their abundance and easy availability in Switzerland, and because they – or closely-related species – have previously been used as model systems for the study of spatial organisation in social insects^20,21,23,27,44^. The colony sizes used in our experiments (Table 1) fell within the natural range of sizes experienced by these species in nature, either as recently founded colonies (*L. niger* colonies are founded by a single queen and progressively grow from a few workers to mature sizes of up to 40,000 workers over the course of several years; new honeybee colonies are founded by swarms counting 2400–41,000 bees^61^) or as mature colonies (all colonies of *T. nylanderi* and *L. acervorum* used in our experiments were mature colonies collected whole from the field).

In all species, a paper tag bearing a unique two-dimensional barcode was glued to the thorax of individuals to allow automated tracking of their movements (Fig. S1). In the ants, tagging of all individuals was performed in a single session two days before the beginning of the experiment, whilst in the bees, newly-emerged workers (one-day-old or less) were tagged every 3 days over the 21 days prior to the beginning of the experiment (Supplementary Note 1).

Tagged colonies were kept in glass observation nests with a single entrance (internal nest dimensions, *A. mellifera*: 69 × 45 × 4 cm, *L. niger*: 70 × 40 × 8 mm; *L. acervorum*: 63 × 42 × 2 mm, *T. nylanderi*: 63 × 42 × 1.5 mm). The honeybee observation nests also included a 64 × 44 cm wooden frame enclosing a double-sided wax comb containing honey, pollen, and developing brood^20^. Bees were free to move between both sides of the comb. In all species, individuals were allowed to freely exit and enter the nest. Ants were provided with *ad libitum* food (*Drosophila*, sugar solution) and water in a foraging arena, while bees foraged on natural resources outside. Both the ant and honeybee observation nests were exposed to diurnal cycles of temperature and light (Supplementary Note 1).

High resolution digital video cameras operating at two frames per second were used to identify the location and orientation of each tag across successive images^22^. All colonies were continuously tracked for three days, which corresponded to the inter-cohort time in the honeybee colonies. The trajectories of each worker, and the physical contacts between workers (Fig. S18 and Supplementary Note 14) were extracted using an existing software pipeline^62^.

### Building bipartite site-visit networks

To quantify the spatial preferences of individual ants and bees, the interior of the nest was discretised into a regular hexagonal lattice (Fig. 1a, b). Because the worker body lengths of our four study species span an order of magnitude (from ~ 1.5 mm for *T. nylanderi* to ~ 15 mm for *A. mellifera*), the width of the hexagonal bins were defined as 1/4 of the mean worker body-length.

To characterise the spatial preferences of different individuals to different parts of the nest, we counted the number of times ${n}_{i}^{s}$ that each individual *i* visited each hexagonal site *s*. A visit by individual *i* to site *s* began when *i* crossed the border into *s*, and was terminated when *i* crossed the border out of *s*, regardless of the amount of time spent inside. To prevent stationary individuals located on the border between two adjacent sites from rapidly accumulating many single-frame visits to the two sites, successive visits to a same site were only counted when at least 20s elapsed between the end of the previous visit and the start of the next.

The site-visit data were used to construct a bipartite network, in which individuals (layer 1) were connected by undirected edges to the sites (layer 2) they visited (Fig. 1c, d). Because individuals typically made multiple visits to the same sites, each edge *i*–*s* was weighted according to the total number of times individual *i* visited site *s*, that is, ${n}_{i}^{s}$.

### Partitioning site-visit networks into modules

The extent to which the site-visit networks were partitioned into discrete ‘modules’ (i.e., set of workers with similar space-use patterns and the set of sites that they exhibit strong ties to) was assessed using the DIRTLPAwb+ algorithm for partitioning weighted bipartite networks^39^. This algorithm searches for the partition that maximises the number and strength of the links within modules, whilst minimizing connections between modules. The number of modules was not specified a priori by the user, but was identified by the algorithm. All site-visit networks had positive modularity (Fig. S3), indicating that they could be partitioned into a set of well-separated modules (Figs. 1e–h, S2, and S4–S5). The modules in each partition were then assigned functional labels according to the following rules. First, the module whose sites were on average closest to the nest entrance was labelled ‘forager’ module. Second, the module or modules with the greatest spatial overlap with the brood pile in the ant colonies or the broodnest(s) in the honeybee colonies were labelled ‘nurse’ module(s). After defining the forager and nurse modules, the remaining modules (if any) were labelled as follows. If there was only one module remaining after identifying the nurse and forager modules, as was typically the case in honeybee colonies, it was labelled ‘peripheral’. If there were two modules remaining, as was typically the case in ant colonies, then the module whose sites were on average closer to the nest borders (i.e., to the periphery of the nest) was labelled ‘peripheral’, and the remaining module labelled ‘intermediate’. In some cases, the DIRTLPAwb+ algorithm identified five or more modules (9.0% of all iterations across all species and colonies). In these cases, the supernumerary modules never contained more than 1 or 2 individuals, and as they could not be unequivocally assigned using our labelling scheme, they were left unclassified for these iterations.

### Validating network modules

As a network constructed by a purely random process could exhibit apparent modular structure by chance, we tested whether the discovered modules represent statistically significant entities. To do so, we produced 1000 null model random networks for each observed network using an established permutation method for bipartite networks^63^ (Supplementary Note 2). Comparisons between the maximum modularity of the observed networks with that of the corresponding random networks showed that, in all four species, the observed modularity was significantly greater than expected by chance (Fig. S3).

### Constructing worker task profiles

A unique labour profile for each ant and each honeybee was constructed by estimating the activity of each worker in the following four tasks:

1. Entrance guarding: workers were classed as guarding when they were (i) within two body lengths of the entrance, (ii) roughly facing the entrance, i.e., with a body alignment diverging from the direct heading to the entrance by no more than *π*/2 radians, and (iii) ‘on station’ at the entrance, as defined by trajectory coordinates with an associated first passage time (ref. 64; time taken for the individual to pass beyond a circle centred on its current location with a radius of two body-lengths) in excess of 500s.

2. Patrolling: workers were classed as patrolling^65^ when they were (i) active, and (ii) ‘roaming’, as defined by first passage times of <5min for a circle with a radius of four body-lengths.

3. Queen attendance: workers were classed as attending the queen if they were in physical contact and facing towards the queen, as defined by the trapezoid method for identifying contacts described in Supplementary Note 14.

4. Foraging: workers were defined as foraging when they left the nest and entered the foraging arena.

As the total time an individual allocates to a given task, and the number of times it performs that task can vary (nearly) independently, we quantified both the total time spent on each task, and also the number of bouts of each task. As individuals were occasionally lost from view all measures were normalized by the total number of trajectory fixes for a given individual.

### Assigning module scores for individuals and sites

As discrete categories are not always suitable to describe continuous biological processes, we developed a methodological extension that allows for overlapping modules. To do so, we exploited the stochastic nature of the DIRTLPAwb+ algorithm and applied it to each network 1000 times, thus producing an ensemble of slightly different partitions. After eliminating duplicate partitions, we defined the score denoting the membership of each individual *i* (or site *s*) to each module *M* as the proportion of partitions in which *i* (or *s*) was assigned to *M*. Thus, each individual and each site was assigned a set of module scores summing to 1 (Supplementary Note 2).

### Measuring diversity

We used an information-theoretic approach to obtain three measures of the heterogeneity of the module scores for individuals and spatial locations. These were termed the module score *diversity* of (i) the visitors to a given site, (ii) the sites an individual visited, and (iii) the nestmates an individual contacted. To do so, we first defined the typical module score profile of (i) the typical visitor to a given site, (ii) the typical site an individual visited, and (iii) the typical nestmate an individual contacted by calculating the following weighted averages:

$${Typical}\,{visitor:}\begin{array}{l}{\left[\begin{array}{c}{N}^{*}\\ {I}^{*}\\ {P}^{*}\\ {F}^{*}\end{array}\right]}_{visitor,s}=\frac{\mathop{\sum }\limits_{i\in colony}^{}{n}_{i,s}\cdot {\left[\begin{array}{c}N\\ I\\ P\\ F\end{array}\right]}_{i}}{\mathop{\sum }\limits_{i\in colony}^{}{n}_{i,s}}\\ \end{array}\,\,;$$

$${Typical}\,{visited}\,{site:}\begin{array}{l}{\left[\begin{array}{c}{N}^{*}\\ {I}^{*}\\ {P}^{*}\\ {F}^{*}\end{array}\right]}_{visited,i}=\frac{\mathop{\sum }\limits_{s\in nest}^{}{n}_{i,s}\cdot {\left[\begin{array}{c}N\\ I\\ P\\ F\end{array}\right]}_{s}}{\mathop{\sum }\limits_{s\in nest}^{}{n}_{i,s}}\\ \end{array}\,\,;$$

$${Typical}\,{contacted}\,{nestmate:}\begin{array}{l}{\left[\begin{array}{c}{N}^{*}\\ {I}^{*}\\ {P}^{*}\\ {F}^{*}\end{array}\right]}_{contacted,i}=\frac{\mathop{\sum }\limits_{j\in colony,j\ne i}^{}{c}_{i,j}\cdot {\left[\begin{array}{c}N\\ I\\ P\\ F\end{array}\right]}_{j}}{\mathop{\sum }\limits_{j\in colony,j\ne i}^{}{c}_{i,j}}\\ \end{array}$$

where [N^*^, I^*^, P^*^, F^*^] denotes the module score profile of (i) the typical visitor of a given site *s*, (ii) the typical site visited by an individual *i*, or (iii) the typical nestmate contacted by individual *i*; *n*_*i*,*s*_ denotes the number of visits by individual *i* to site *s*; *c*_*i*,*j*_ denotes the number of contacts between individuals *i* and *j*; [*N*, *I*, *P*, *F*] denotes the scores of (i) individual *i*, (ii) site *s*, or (iii) individual *j* for the Nurse, Intermediate, Peripheral and Forager modules. Because these are weighted averages, there is a proportionally greater contribution to the typical score profile by (i) individuals that visit site *s* more frequently, (ii) sites that individual *i* visits more frequently and (iii) individuals that individual *i* contacts more frequently. These typical module score profiles were then used to calculate the module score diversity *D* of each site *s* and individual *i*;

$${{{{{{{{\rm{D}}}}}}}}}_{visitor,s}=\frac{{{{{{{{{\rm{H}}}}}}}}}_{visitor,s}}{{{{{{{{{\rm{H}}}}}}}}}_{\max }};\,{{{{{{{{\rm{D}}}}}}}}}_{visited,i}=\frac{{{{{{{{{\rm{H}}}}}}}}}_{visited,i}}{{{{{{{{{\rm{H}}}}}}}}}_{\max }};\,{{{{{{{{\rm{D}}}}}}}}}_{contacted,i}=\frac{{{{{{{{{\rm{H}}}}}}}}}_{contacted,i}}{{{{{{{{{\rm{H}}}}}}}}}_{\max }},$$

where H_*visitor*,*s*_ is the entropy of the typical scores of the typical visitor to site *s*, ${[{N}^{*},{I}^{*},{P}^{*},{F}^{*}]}_{visitor,s}$; H_*visited*,*i*_ the entropy of the typical scores of the typical site visited by individual *i*, ${[{N}^{*},{I}^{*},{P}^{*},{F}^{*}]}_{visited,i}$; and H_*contacted*,*i*_ the entropy of the typical scores of the typical nestmate contacted by individual *i*, ${[{N}^{*},{I}^{*},{P}^{*},{F}^{*}]}_{contacted,i}$. The diversity index ranges from 0 (e.g., sites that are only visited by specialists for one module – individuals that scored 1 for one module and 0 for all other modules) to 1 (e.g., sites that are visited by module generalists and/or by an equal mix of specialists from all modules).

### Module score similarity between workers and sites

The similarity between the module scores of a worker and a site that it visits was quantified using cosine similarity, a standard measure of the distance between two vectors, defined as the cosine of the angle between them. Each node was represented by the vector of its module scores, and the cosine similarity of individual *i* and site *s*, was defined as follows:

$${\cos }_{i,s}=\frac{\mathop{\sum }\limits_{M\in (N,I,P,F)}^{}{M}_{i}\times {M}_{s}}{\sqrt{\mathop{\sum }\limits_{M\in (N,I,P,F)}^{}{{M}_{i}}^{2}}\sqrt{\mathop{\sum }\limits_{M\in (N,I,P,F)}^{}{{M}_{s}}^{2}}},$$

where *M*_*i*_ and *M*_*s*_ are the module scores of individual *i* and site *s* for module *M*, respectively. Because all module scores were positive, in our study cosine similarity ranged from 0 (orthogonal vectors) to 1 (identical vectors).

### Measuring long-range attraction

To assess whether long-range attraction of workers towards their primary module could be a mechanism for producing spatial segregation between workers, we defined a taxis index T_*M*,*i*,*s*_, which measures the attraction of an individual *i* to a focal module *M*, at each site *s* that it visited. The taxis index was defined by calculating the projection of the mean resultant vector of all trajectory segments of *i* starting at site *s*, $\overrightarrow{{v}_{i,s}}$, onto a vector pointing directly from site *s* to the nearest point on the boundary of the focal module *M* (Fig. S11). Thus, positive values of T_*M*,*i*,*s*_ occur when an individual exhibits a tendency to head toward *M*, and negative values indicate a tendency to move away from *M*.

To test whether individuals display greater attraction towards their primary module than individuals belonging to other modules, we then defined, for each module *M* and each site *s* lying outside *M*, a mean taxis index $\overline{{{{{{{{{\rm{T}}}}}}}}}_{M,s}}$ for either ‘resident’ workers (i.e., individuals whose primary module is *M*) or ‘non-resident’ workers (i.e., individuals whose primary module is not *M*). For each species, we then tested whether worker ‘residency’ (resident vs. nonresident) had a significant effect on the mean taxis index $\overline{{{{{{{{{\rm{T}}}}}}}}}_{M,s}}$ at different distances from the module boundary using generalised mixed-effect models with $\overline{{{{{{{{{\rm{T}}}}}}}}}_{M,s}}$ as a dependent variable, worker group and colony size as fixed effects, and colony identity, site identity and focal module identity as random effects. As this analysis involved multiple testing (one for each distance), *p*-values were adjusted using the Benjamini-Hochberg (BH) correction for multiple testing.

### Investigating location-dependent movement

We here describe three fundamental measures of individual locomotion. The first was the site-specific activity probability, which describes the likelihood that a particular individual is in motion at a particular site. The activity probability was obtained by first decomposing each trajectory into an alternating sequence of active and inactive bouts using a combination of change-point analysis^6^ and cluster analysis (Fig. S19, Supplementary Note 15). For each individual *i* – site *s* pair, we then calculated the activity probability P_*i*,*s*_, that is, the proportion of time that *i* was in the active state when visiting site *s* (Fig. S20 and Supplementary Note 16).

To further characterise locomotion while individuals were in the active state, we defined two additional measures for every site *s*, visited by each individual *i*, namely the mean speed while active v_*i*,*s*_, and the mean unsigned turn angle while active *θ*_*i*,*s*_ (Fig. S20). Calculating these measures using only trajectory coordinates associated with active individuals ensured that these measures capture properties of movement, rather than the probability of moving.

### Mapping module gradient fields

To estimate the overall module gradient field for a given module *M*, we performed a spatial multiple regression at each site *s*. To do so, we first defined a local neighbourhood around each site *s*, which included all sites whose centres were within one worker body length of the centre of the focal site. The scores for module *M* were then regressed on the x- and y-coordinates of the centres of all sites in this neighbourhood. This allowed us to extract the equation of best fit for the scores for module *M* around each site *s*, that is, *M*_*s*_ = *a*_*M*,s_*x + *b*_*M*,s_*y + *c*_*M*,*s*_. The coefficients of this equation were then used to define a scalar vector, $${\overrightarrow{{{{{{{{\rm{g}}}}}}}}}}_{M,s}=\left[\begin{array}{c}{a}_{M,s}\\ {b}_{M,s}\end{array}\right]$$, whose directional component corresponded to the direction of steepest increase in the scores of module *M* around *s*, and whose magnitude component ∣*g*∣_*M*,*s*_ represents the steepness of that increase. Neighbourhoods in which all sites had the exact same score for module *M* had a magnitude of 0 and an undefined direction. Finally, the local vectors for all sites were combined to produce a two-dimensional gradient field ${\overrightarrow{{{{{{{{\rm{F}}}}}}}}}}_{M}$ for module *M* across the entire nest. The gradient field typically took values of 0 at the core or far outside the module of interest, where module scores tended to take homogeneous values of 1 (module core) or of 0 (outside of the module). By contrast, the gradient field typically took non-zero values in areas of transition between adjacent modules, with the steepest values coinciding with the module’s borders (Fig. 8b).

### Quantifying worker movement in the module gradient field

As worker movement may be affected by spatial heterogeneities and physical features within the nest, such as the presence of physical barriers like nest walls, we first established the typical movement of the average worker within the gradient field of each module. To do so, for each module *M* and each individual *i*, each trajectory segment was classified into one of two categories according to whether the individual was heading up-gradient or down-gradient within that module’s gradient field, ${\overrightarrow{{{{{{{{\rm{F}}}}}}}}}}_{M}$. Segments were classified as up-gradient when the absolute angular difference between the trajectory heading and the gradient vector direction at that location was smaller than *π*/2 radians, and down-gradient when it was greater than *π*/2 radians.

To quantify the turning behaviour of individuals heading down-gradient in the field of their primary module (i.e., when *i* is heading out of *M*_*i*_), we defined a ‘relative down-gradient turn angle’,

$${{\Delta }}{\theta }_{i,s}^{{{{{{{{\rm{down}}}}}}}}}={\bar{\theta }}_{i,s,{M}_{i}}^{down}-{\bar{\theta }}_{s,{M}_{i}}^{down}$$

where ${\bar{\theta }}_{i,s,{M}_{i}}^{down}$ is the mean turn angle of individual *i* when heading down-gradient in its primary module field, ${\overrightarrow{{{{{{{{\rm{F}}}}}}}}}}_{{M}_{i}}$, at site *s*, and the second term, ${\bar{\theta }}_{s,{M}_{i}}^{down}$ is the mean turn angle of all individuals heading down-gradient in ${\overrightarrow{{{{{{{{\rm{F}}}}}}}}}}_{{M}_{i}}$ at site *s*. Positive values of ${{\Delta }}{\theta }_{i,s,M}^{{{{{{{{\rm{down}}}}}}}}}$ occur when a resident worker approaching the border of its primary module from the inside makes bigger turns than the average worker. Such workers tend to turn away from the border, and so are likely stay inside their primary module.

To quantify the behaviour of individuals heading up-gradient in their primary module (i.e., *i* heading into *M*_*i*_), we also defined the ‘relative up-gradient turn angle’,

$${{\Delta }}{\theta }_{i,s}^{{{{{{{{\rm{up}}}}}}}}}={\bar{\theta }}_{i,s,{M}_{i}}^{up}-{\bar{\theta }}_{s,{M}_{i}}^{up}$$

Negative﻿ ﻿values of ${{\Delta }}{\theta }_{i,s}^{{{{{{{{\rm{up}}}}}}}}}$ occur when a resident worker heading into *M* from the outside turns less than the average worker. Such workers tend not to turn away from the border, and so are likely to enter their primary module.

### Statistical analyses

The proportion of specialist workers and of non-overlapping sites were analysed using general linear models (GLM) implemented using the package *stats* version 3.6.1 for *R*. The proportion of specialist workers was subjected to a square-root transformation to ensure normality of residuals (Shapiro–Wilk test, *n* = 50, proportion of specialist workers: *W* = 0.987, *p* = 0.84; proportion of non-overlapping sites: *W* = 0.980, *p* = 0.53). After fitting the GLM, the significance of the main effects was evaluated using F-tests.

All linear mixed-effects models (LME) and the Poisson generalized linear mixed-effects model (GLMM) were implemented using the package *lme4* version 1.1–13^66^ for *R*. In these models, continuous explanatory variables were scaled where necessary (e.g., in models including both colony size and individual-site similarity as explanatory variables, as these differed in scale by several orders of magnitude), though scaling did not affect the direction or significance of the main effects. To check that the LME model assumptions were not violated, we did not use traditional normality tests because those are not suitable for large sample sizes of more than 300 data points^67^. Instead, we calculated the skewness and kurtosis of each model’s residuals and checked that they were compatible with a normal distribution (i.e., skewness between −2.1 and +2.1 and kurtosis <7.1;^67^). Where necessary, dependent variables were subjected to a square root-, power- or log-transformation to ensure the normality of residuals. In the final models, skewness ranged from −2.1 to 0.5 and kurtosis from 1.6 to 6.6. After fitting the mixed-effects models, the significance of main effects was evaluated using Wald *χ*^2^-tests. All post-hoc comparisons were implemented by the package *multcomp* version 1.4-10^68^ for *R* using the BH method to correct for multiple testing.

### Reporting summary

Further information on research design is available in the Nature Portfolio Reporting Summary linked to this article.

## Supplementary information

Supplementary Information

Description of Additional Supplementary Files

Supplementary Movie 1

Reporting Summary

## Data Availability

Source data are provided with this paper. The automated tracking datasets (raw trajectories) generated for the current study are available in the Dryad repository 10.5061/dryad.9w0vt4bjb.

## Source data

Source Data

## Code availability

Code for constructing the bipartite spatial networks, and for the simulations, are available in the Zenodo repository, 10.5281/zenodo.6787674.
