# Supplementary material for: Two simple movement mechanisms for spatial division of labour in social insects
Source: Nat Commun. 2022 Nov 15;13:6985. doi: 10.1038/s41467-022-34706-7 (PMC9666475; doi:10.1038/s41467-022-34706-7)
Supplement: Supplementary file 1 — Supplementary Information [file 41467_2022_34706_MOESM1_ESM.pdf]

Two simple movement rules for spatial division of labour in social insects: Supplementary Information

Thomas O. Richardson[†], Nathalie Stroeymeyt[†], Alessandro Crespi, Laurent Keller

[†] These authors contributed equally to this work.

Supplementary Note 1: Experiments

In the ant colonies, a unique ARtag barcode (Fiala, 2005) printed on synthetic polymer paper was affixed to each worker. In the honeybee colonies, workers were tagged using AprilTag barcodes (36h10 family, Olson, 2011), as this library provided a larger number of tags required for the much larger honey bee colonies. Tags were affixed to the dorsal thorax using a small drop of adhesive (Pattex Power Easy Gel) after the individual had been temporarily immobilized by chilling on ice for the honey bees, or brief immersion in a carbon dioxide bath for the ants (Fig. S1). The dimensions of the tags used for each species were varied according to body size (*A. mellifera*; 1.86×1.86 mm, *L. niger* & *L. acervorum*; 0.7×0.7 mm, *T. nylanderi*; 0.5×0.5 mm).

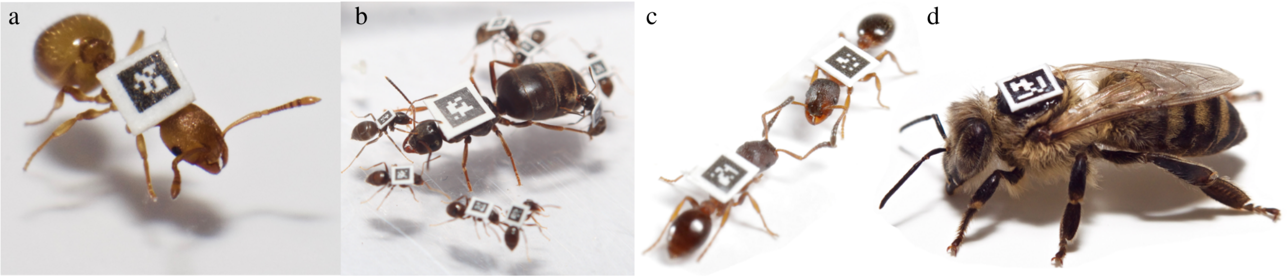

Figure S1: **Tagged ants and bees.** (**a**) *T. nylanderi.* (**b**) *L. niger*, (**c**) *L. acervorum*, (**d**) *A. mellifera*

Automated tracking was performed under infra-red light, to which both ants and bees are insensitive (Briscoe and Chittka, 2001). Image capture was perfomed by high-resolution, infra-red sensitive tracking cameras (ants: Vector international FCi4-14000, 4560×3048 pixels; bees: Vieworks VA-29M, 6576×4384 pixels) at a rate of two frames per second. Each camera was connected to an array of infra-red LEDs whose flashes coincided with the camera exposure, allowing the detection of tags even in the absence of visible light(Mersch et al., 2013).

In all three ant species, colonies were housed in a rectangular nest (*L. niger*: 70x40x6mm; *L. acervorum*: 63x42x2mm; *T. nylanderi*: 63x42x1.5mm) covered with an infra-red transmitting filter glass slide (R-64, Hoya, USA). The filter blocked most visible light and only allowed wavelengths longer than 640nm to pass through, which ensured that the nest interior appeared dark to the ants while allowing tracking inside. The nest was connected to a foraging arena, which the ants could access freely thoughout the experiment, and which contained *ad libitum* protein (*Drosophila*), sugar water, and drinking water. Twelve hours prior to the start of the video tracking, the experimental arena was placed within a foam tracking box containing one tracking camera positioned above the nest. The temperature, humidity, and visible light regimes were tightly controlled within the tracking box. During the day (07:00-19:00) the tracking box was illuminated with visible light and the temperature was set to 26°C, whilst at night (19:00-07:00) there was no visible light, and the box was cooled to 22°C. Humidity was maintained at 60% at all times.

The honey bee colonies were housed in modified observation nests, consisting of a single continuous wax comb (50×75 cm) that was sandwiched between two glass walls (Seeley, 1982). A 10mm gap between the comb suface and the inner surgace of the glass wall was left to allow the bees to move unhindered across the comb. Each observation nest was placed inside a sheltered housing situated outside the lab. Each housing consisted of a large rectangular box (1×1×2.5 m) in which two tracking cameras, placed at opposite ends of the box, recorded the movements of bees on either sides of the wax comb through the glass walls. To reproduce the natural inside-nest conditions, the housings were closed to prevent the incursion of natural light. Two fans were installed at each end of the enclosure to remove excess heat buildup from the infra red LEDs.

Worker age data were gathered for the honey bee *A. mellifera* and the ant *L. niger*. For *L. niger*, age data were acquired by the application of coloured paint marks. Every two weeks each colony was inspected and all unmarked ants removed. A unique combination of two coloured paint marks encoding the inspection date was applied to the head of each unmarked ant, which was then returned to its natal nest. For all *L. niger*

colonies, weekly marking was conducted for 11 months, producing an age-structured population of marked workers ranging between 7 and 343 days old, with a mean of 66 days (S.E.=4.0 days, N=20 colonies).

In *A. mellifera* age data were acquired by introducing successive cohorts of tagged callow workers of known age (Seeley, 1982; Johnson, 2008; Baracchi and Cini, 2014). A cohort of callow tagged workers was produced by removing a frame containing mature brood from the broodnest of a donor colony, and incubating this frame overnight at 34.5^{o}C. The next day, 290 newly-eclosed workers were harvested from the frame, and an AprilTag barcode affixed to each. The tagged cohort was then immediately introduced into the observation nest. Each colony was subjected to eight cohort introductions, with a 3 days interval between successive introductions. This produced an age-structured population of tagged workers ranging between 1 and 22 days old, with a mean age of 8.4 days (S.E.=0.3 days, N=10 colonies).

Supplementary Note 2: Elevated modularity in site-visit networks

Visual inspection of the bipartite site-visit networks indicated the presence of non-random structure in the connections between individuals and sites (Fig. S2). This was confirmed by analysing these networks with a stochastic community detection algorithm, the DIRTLPAwb+ module detection algorithm (Beckett, 2016), which indicated that each network appeared to be segregated into multiple modules. However, as a network constructed by a purely random process can exhibit apparent structure just by chance, we tested whether the discovered modules represent statistically significant entities. To that end, each observed site-visit network was repeatedly permuted using the fixed-rows, fixed-columns null model of Connor and Simberloff (1979), thus generating an ensemble of random networks that lacked modular structure but which preserved important statistical properties of the original, such as the number of site visits made by each individual and received by each site. This null model generates bipartite networks in which the edges are randomly redistributed among all node pairs, whilst obeying the constraint that the observed weighted degree of each node is are preserved, which corresponds to the preservation of the marginal row and column totals in the (weighted) bipartite adjacency matrix. In the resulting null networks, ants and sites interact randomly, whilst allowing (i) sites to vary in popularity among the ants, and (ii) ants to vary in their site visitation activity across all sites. Implementation of the fixed-rows, fixed-columns permutation null models was performed using the Patefield algorithm (Patefield, 1981), as implemented by the *r2dtable* function for *R* version 3.3.2 (R Core Team, 2016), and was repeated 100 times per network.

The presence of modular structure in the original and permuted networks was then assessed using the number of modules, the weighted modularity Q_W (Dormann and Strauss, 2014), and the realized modularity Q_R (Poisot, 2013; Beckett, 2016) produced by running the DIRTLPAwb+ module detection algorithm over each network. Comparisons between the original and permuted modularity metrics revealed that in all four species the number of modules (Fig. S3a), the observed modularity Q_W (Fig. S3b), and the observed realized modularity Q_R (Fig. S3c) were significantly greater than that expected by chance (Fig. S3b), hence the modules are real entities, and not statistical artefacts.

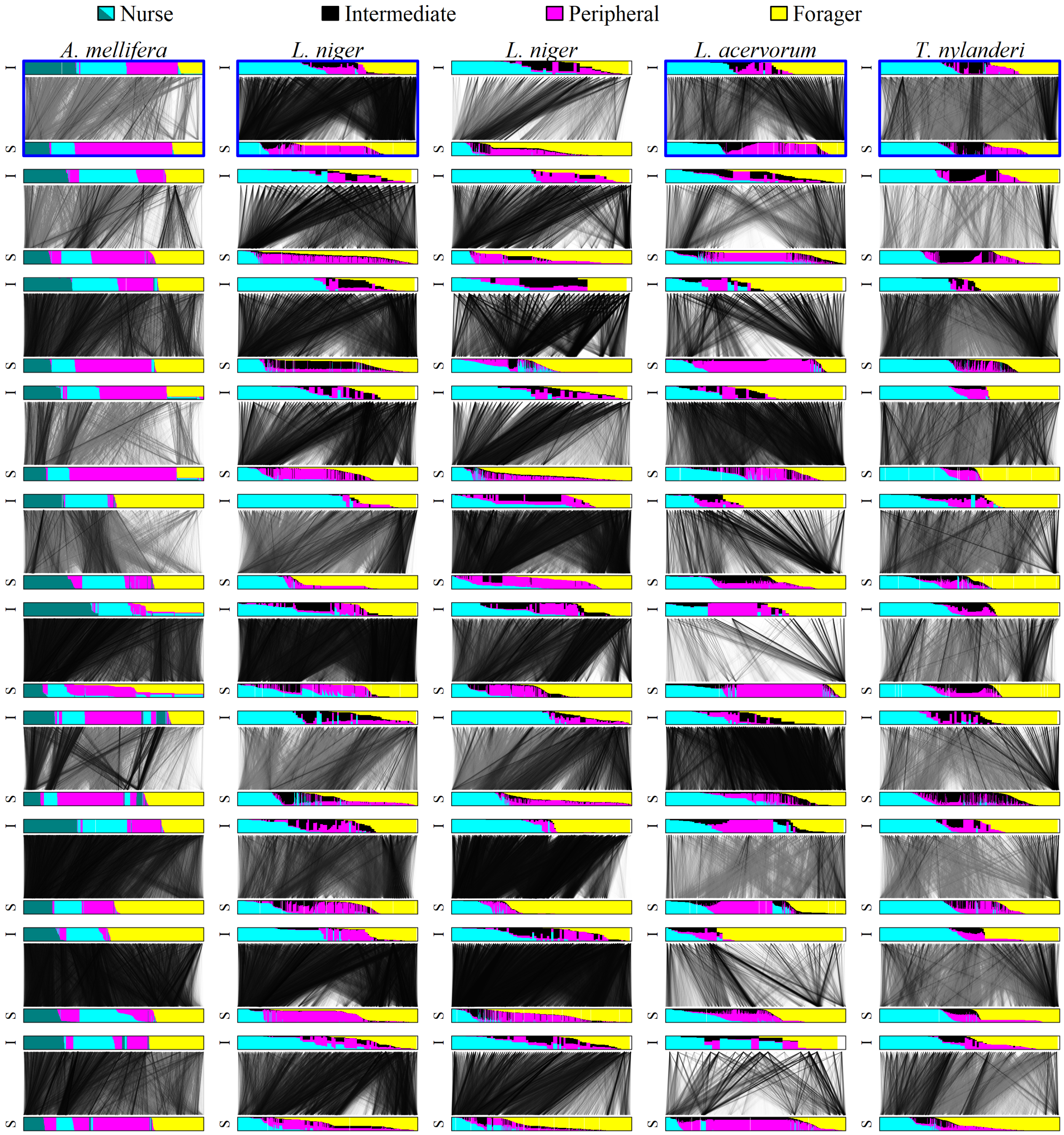

Figure S2: **Bipartite site-visit networks.** Edges connect individuals (I) to the sites (S) they visit. Edge thickness is proportional to the number of visits. The coloured bars indicate the module scores for each node (cyan; nurse, magenta; peripheral, yellow; forager, black; intermediate). In *Apis mellifera* the two shades of cyan indicate the two nurse modules. The four colonies framed in blue in the top row correspond to the colonies used as an illustration in the main text.

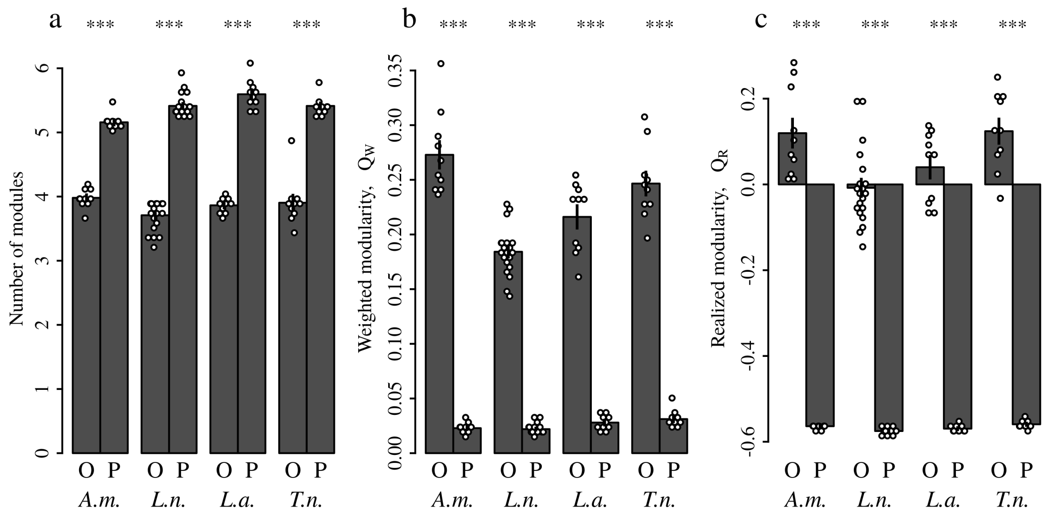

Figure S3: **Elevated modularity in the site-visit networks.** Barplots show three alternative measures of network structure in the original networks (O; 1000 iterations of the DIRTLPAwb+ algorithm for each observed network), and the permuted networks (P; 100 permuted networks per observed network; 1 iteration of the DIRTLPAwb+ algorithm per permuted network). **(a)** The number of modules in the network. **(b)** The weighted modularity, Q_W **(c)** The realized modularity, Q_R. Bars represent across-colony grand means and whiskers the associated standard errors, hence each colony contributes a single value to each bar (shown as white points). Colony means were calculated across the unique partitions for each colony. Asterisks indicate results of two-tailed paired t-tests (***; p<0.0001). A.m.: *A. mellifera*, L.n.: *L. niger*, L.a.: *L. acervorum*, T.n.: *T. nylanderi*. Analyses were performed on mean values for n = 50 original and n = 50 permuted networks. Source data are provided as a Source Data file.

Supplementary Note 3: Module scores for individuals and sites

To acquire a set of module scores that quantify the extent to which a given network node belongs to a given module, the DIRTLPAwb+ module detection algorithm (Beckett, 2016) was applied 1000 times to each of the 50 bipartite networks (Fig. S2). The modules in each partition were then assigned categorical behavioural task labels according to the procedure described in the main paper. As a given partition was sometimes discovered more than once, duplicates were discarded, resulting an ensemble of <1000 *unique* partitions for each network.

The extent to which given individual i or site s belongs to a particular module M in the site-visit network (where $M \in$ {Nurse, Intermediate, Peripheral, Forager}), is given by the module score,

$$\mathrm{M}_i = \frac{\mathrm{n}_{i,M}}{\mathrm{n}} \quad \text{and} \quad \mathrm{M}_s = \frac{\mathrm{n}_{s,M}}{\mathrm{n}}$$

where $\mathrm{n}_{i,M}$ and $\mathrm{n}_{s,M}$ are the number of solutions in the ensemble of partitions that assign nodes i and s to module M, and n is the total number of solutions in the ensemble of partitions for the site-visit network.

Fig. S4 shows the spatial distribution of the site scores for each module in an example *T. nylanderi* colony. A different colour was assigned to each module (Nurse: cyan, Intermediate: black, Peripheral: magenta, Forager: yellow), and transparency was used to quantify the value of each site score. Module-specific maps were then overlaid to produce a global map showing the relative distribution and overlap of all modules. Figure S5 shows the module maps for each colony.

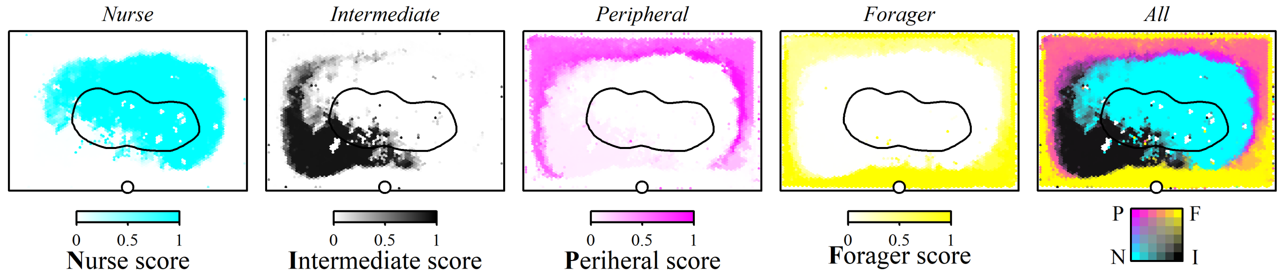

Figure S4: **Spatial distribution of site module scores in an example *T. nylanderi* colony.** The first four maps represent the spatial distribution of the site scores for each module (*Nurse*: cyan (C), *Intermediate*: black (K), *Peripheral*: magenta (M), *Forager*: yellow (Y)). Transparency was used to quantify the value of each site score, so that a site which has a score of 1 for a given module is opaque while a site which has a score of 0 for a given module is completely transparent. The four maps are then overlaid to produce a combined map (*All*) where each unique CMYK combination represent a particular set of module scores.

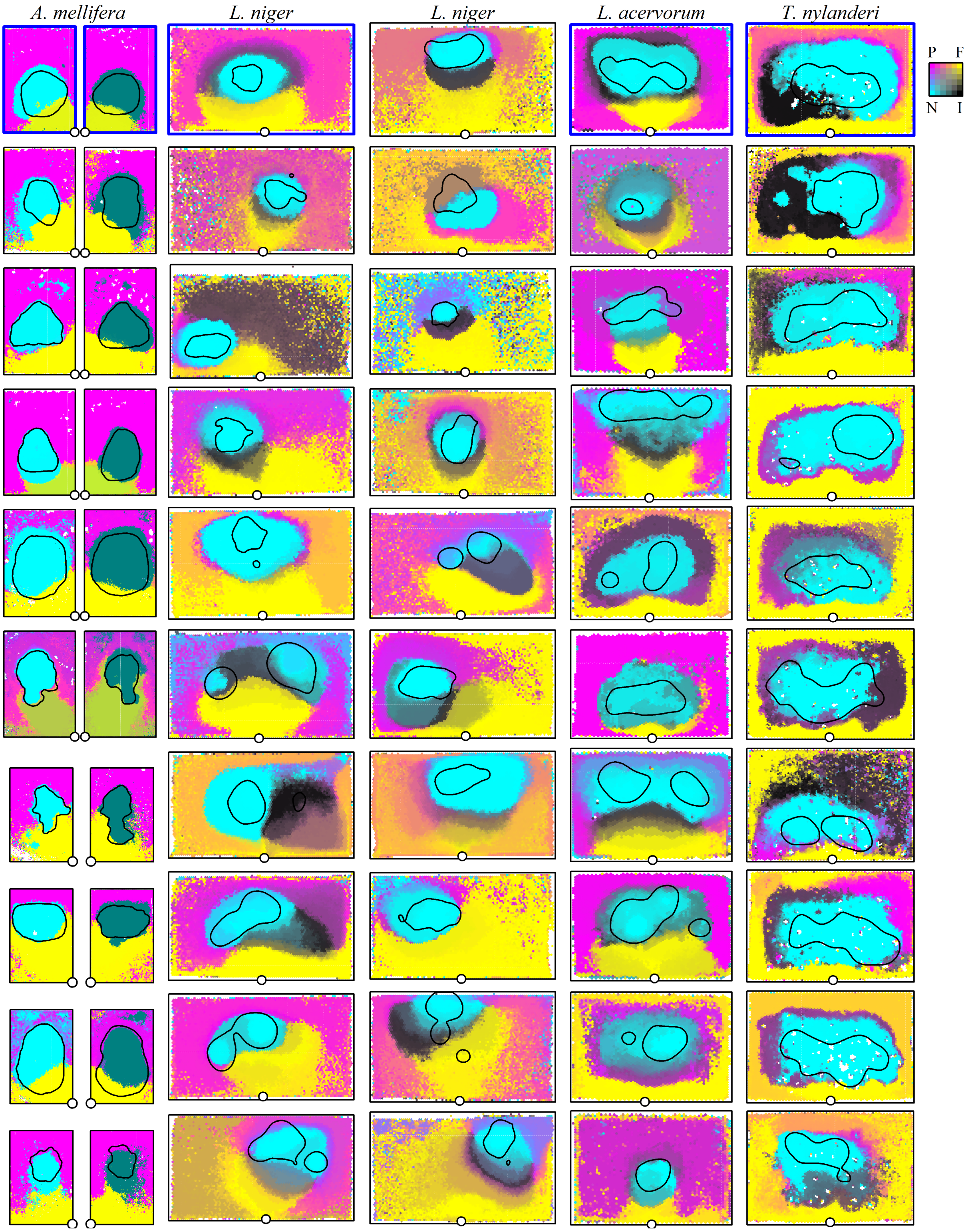

Figure S5: **Spatial module maps.** Sites are coloured according to the linear (subtractive) combination of the colours for each module, weighted by its module scores (CMYK colour space), as in Figure 2; thus a site with a forager score of 1 (‘pure’ forager site) is shown in yellow (top right corner in the square chart), while a site with a forager score of 0.5 and a peripheral score of 0.5 is shown in orange (middle of top row in the square chart). In the honeybee colony, two different shades of cyan are used to depict the two distinct nurse modules. Solid black lines indicate the borders of the broodnest in *A. mellifera*, and the outline the brood pile in the ants. The circle indicates the nest entrance. White grid cells correspond to unvisited sites (typically occupied by clusters of brood in the ants). The four colonies framed in blue in the top row correspond to the colonies used as an illustration in the main text.

Supplementary Note 4: Distribution of individuals and sites across modules

In this section we present between-species comparisons of the distribution of individuals and sites are distributed across modules. To do so the primary module of each individual and each site was first identified, which is simply the identity of the module that a given node achieved its highest module score. Finally, for each species the proportion of all individuals or sites with a given primary module are calculated (Table S1).

	**Proportion of sites**				**Proportion of workers**			
	Apis	*Lasius*	*Lepto.*	*Temno.*	*Apis*	*Lasius*	*Lepto.*	*Temno.*
**Nurse**	0.32±0.10	0.19±0.07	0.29±0.11	0.34±0.07	0.50±0.07	0.43±0.10	0.30±0.11	0.39±0.05
**Intermediate**	-	0.085±0.11	0.065±0.07	0.14±0.11	-	0.15±0.12	0.083±0.07	0.11±0.08
**Peripheral**	0.39±0.14	0.28±0.18	0.41±0.20	0.15±0.04	0.21±0.12	0.15±0.07	0.17±0.10	0.13±0.04
**Forager**	0.29±0.10	0.45±0.16	0.23±0.11	0.37±0.12	0.29±0.13	0.26±0.05	0.44±0.16	0.38±0.06

Table S1: **Distribution of sites and workers across modules.** Proportion of nest sites and workers whose primary module corresponds to each of the four identified modules (mean ± standard deviation, calculated across all colonies for each species). Source data are provided as a Source Data file.

Supplementary Note 5: Testing for an association between module membership and task profile

To establish whether the task profile of a worker ant or honey bee can be predicted from its module membership, we used multiple analysis of variance (MANOVA) to test for statistical differences in the task profiles of individuals with different primary modules, where an individual's primary module is defined as the module for which the individual scored highest (Fig. S6).

In all species, the task profiles of individuals that scored highest for the nurse module were significantly different to those that scored highest for the forager module (Fig. S6). In all ants, the task profiles of nurses and foragers was significantly different to that of the intermediates. Furthermore, the task profile of the intermediates was significantly different to that of the peripherals in *L. niger* and *L. acervorum*, though not in *T. nylanderi*. Similarly, the task profile of the peripherals was significantly different to that of the nurses in *L. acervorum* and *T. nylanderi*, though not in *L. niger*. Finally, the task profile of the peripherals was significantly different to that of the foragers in *L. niger* and *T. nylanderi*, though not in *L. acervorum*.

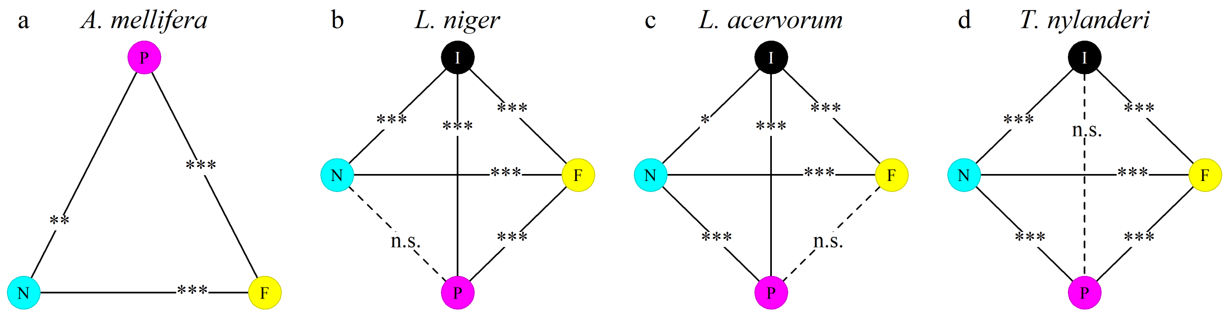

Figure S6: **Workers belonging to different spatial modules exhibit different task profiles.** Networks represent pairwise MANOVA contrasts between the task profiles of individuals with different primary modules in *A. mellifera* (a), *L. niger* (b), *L. acervorum* (c), and *T. nylanderi* (d). Nodes represent modules (N - nurse module, I - intemediate, P - peripheral, F - forager). Edges represent the statistical significance of pairwise post-hoc contrasts with Benjamini-Hochberg correction for multiple testing (n.s.: p >0.05; *: p < 0.05; **: p < 0.01; ***: p < 0.001). Source data are provided as a Source Data file.

Supplementary Note 6: Testing for differences in movement between individuals in different modules

Trajectory analyses of workers that belonged to different primary modules revealed the presence of significant differences in individual movement characteristics between modules (Fig. S7). In the ants, workers that belonged primarily to the forager and peripheral modules were significantly more active, and moved more quickly when active, than those that belonged primarily to the nurse module. In the honeybees, workers that belonged primarily to the forager module also moved significantly more quickly when active than those in the nurse module, but they were significantly less active than the nurses.

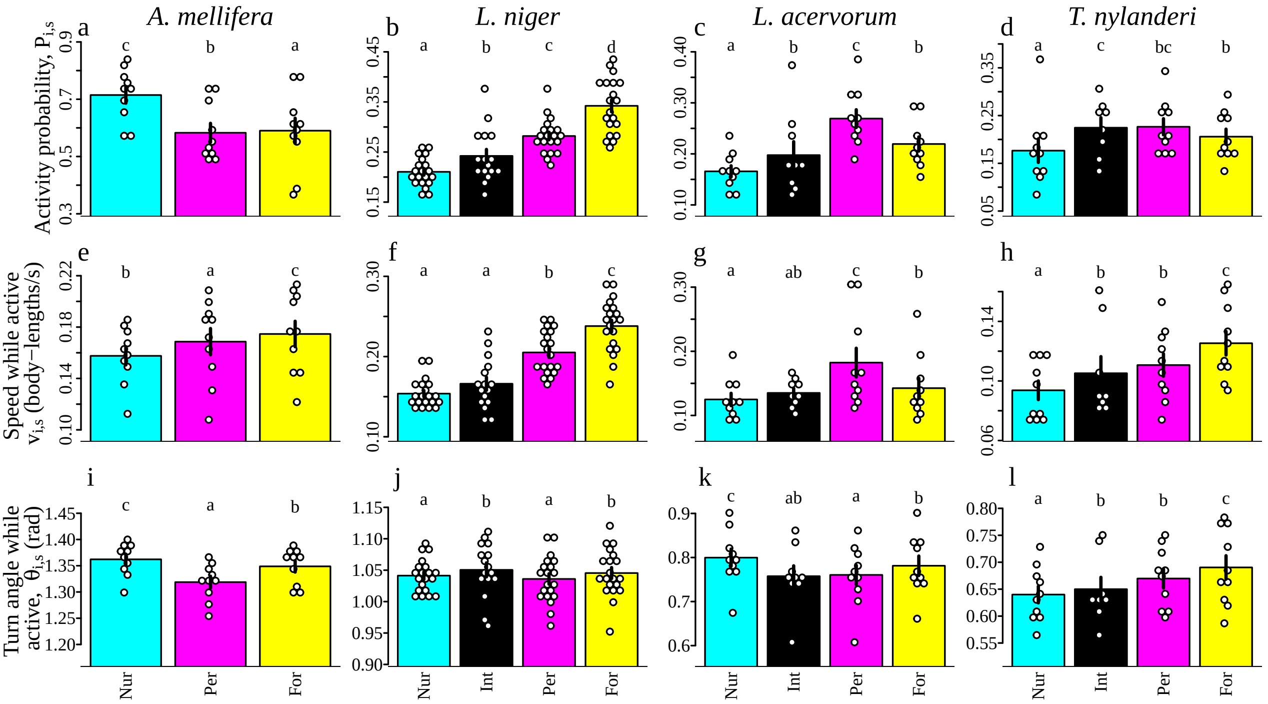

Figure S7: **Workers in different modules display different movement characteristics.** Points, bars & whiskers represent colony means, cross-colony grand means & standard errors of the probability of being in the active state (a-d), the speed of movement while in the active state (e-h) and the absolute turn angle of movement while in the active state (i-l) among individuals from all modules identified in *A. mellifera* (a, e, i), *L. niger* (b, f, j), *L. acervorum* (c, g, k), and *T. nylanderi* (d, h, l). Letters above bars indicate Benjamini-Hochberg corrected post-hoc contrasts (same letters: $p > 0.05$; different letters: $p < 0.05$) between all module pairs in linear mixed-effects models (LME) with colony identity as a random effect, ran separately for each species. To facilitate comparisons between species, the y-axes are scaled to the range for each panel. Analyses based on n = 12 494 workers drawn from n = 50 colonies. Source data are provided as a Source Data file.

Supplementary Note 7: Between-species differences in social and spatial compartmentation

To test whether the degree of module overlap differed between species, we used general linear models (GLM). The GLMs included either the proportion of non-overlapping sites (i.e. proportion of sites with a score of 1 for a module and 0 for all other modules) or the proportion of specialist workers (i.e. proportion of workers with a score of 1 for a module and 0 for all other modules) as dependent variable, and species as main effect. In both models, the proportion of specialist workers was square-root transformed to ensure normality of residuals. Table S2 presents the results of these anayses when the two nurse modules were pooled in the honeybees.

To confirm that these results were not an artefact caused by the pooling of the two honey bee nurse modules, we repeated the analysis above while keeping the two honey bee nurse modules as two distinct modules. Table S3 shows that the results were qualitatively similar to those obtained after pooling the nurse modules.

As it is possible that these differences in social and spatial compartmentation between species could be due to consistent differences in the typical colony size across species (see main text Table 1), we repeated the above analysis using colony size rather than species as the main effect. Predictably (since colony size is associated with species), colony size was also found to have a significant effect on both the proportion of full specialist workers ($F=34.51$, $df=1$, $p<0.0001$) and the proportion of non-overlapping sites ($F=36.65$, $df=1$, $p<0.0001$). However, these models provided a significantly less good fit to the data than those in which the main effect was species

	Proportion non-overlapping sites		Proportion specialist workers	
**Post-hoc comparison**	**t**	**p**	**t**	**p**
L. niger - A. mellifera	-6.28	<0.0001	-6.46	<0.0001
L. acervorum - A. mellifera	-6.34	<0.0001	-5.57	<0.0001
T. nylanderi - A. mellifera	-4.31	<0.0005	-3.80	<0.001
L. acervorum - L. niger	-1.04	0.30	0.03	0.97
T. nylanderi - L. niger	1.31	0.24	2.08	0.065
T. nylanderi - L. acervorum	2.03	0.072	1.77	0.10

Table S2: **Socio-spatial organisation in honey bee colonies is more compartmentalised than in ant colonies (honey bee nurse modules pooled).** Results from pairwise post-hoc tests in which p-values were corrected for multiple testing using the Benjamini-Hochberg method (GLMs, main effect of species on proportion of non-overlapping sites: $F_{3,46}$=16.8, p<0.0001; main effect of species on proportion of specialist workers: $F_{3,46}$=15.7, p<0.0001). All analyses based on n = 50 colonies.

	Proportion non-overlapping sites		Proportion specialist workers	
**Post-hoc comparison**	**t**	**p**	**t**	**p**
L. niger - A. mellifera	-5.85	<0.0001	-5.59	<0.0001
L. acervorum - A. mellifera	-5.95	<0.0001	-4.81	<0.0001
T. nylanderi - A. mellifera	-3.95	<0.001	-3.11	<0.01
L. acervorum - L. niger	-1.02	0.31	0.03	0.97
T. nylanderi - L. niger	1.29	0.25	2.00	0.077
T. nylanderi - L. acervorum	2.00	0.078	1.70	0.11

Table S3: **Socio-spatial organisation in honey bee colonies is more compartmentalised than in ant colonies (honey bee nurse modules separate).** Results from pairwise post-hoc tests in which p-values were corrected for multiple testing using the Benjamini-Hochberg method (GLMs, main effect of species on proportion of non-overlapping sites: $F_{3,46}$=14.7, p<0.0001; main effect of species on proportion of specialist workers: $F_{3,46}$=11.8, p<0.0001). All analyses based on n = 50 colonies.

(Cox test for comparing two non-nested models, improvement of species-models relative to colony size-models, proportion of full specialist workers: z = -3.6, p<0.001; proportion of non-overlapping sites: z = -3.72, p<0.001; improvement of colony size-models relative to species-models, proportion of full specialist workers: z = 0.11, p=0.91; proportion of non-overlapping sites: z = 0.38, p=0.70) .

To further explore whether colony size may have an effect on the degree of social and spatial compartmentation *above and beyond the effect of species*, we fitted additional GLMs including both species and colony size as main effects. These models did not provide a better fit than models which only included species as a main effect (proportion of full specialist workers: F = 0.01, df = 1, p = 0.91; proportion of non-overlapping sites: F = 0.14, df=1, p=0.71).

Finally, we fitted separate GLMs for each species with colony size as a main effect to test for a possible effect of colony size on social and spatial compartmentation *within species*. None of these analyses revealed a significant association between colony size and the proportion of full specialist workers (*A. mellifera*: $F_{1,8}$=0.0097, p=0.92; *L. niger*: $F_{1,18}$=1.08, p=0.31; *L. acervorum*: $F_{1,8}$=0.11, p=0.75; *T. nylanderi*: $F_{1,8}$=0.12, p=0.74) or between colony size and the proportion of non-overlapping sites (*A. mellifera*: $F_{1,8}$=0.06, p=0.81; *L. niger*: $F_{1,18}$=1.42, p=0.25; *L. acervorum*: $F_{1,8}$=2.39, p=0.16; *T. nylanderi*: $F_{1,8}$=1.12, p=0.32).

Supplementary Note 8: Assessing the effect of network size upon the stochasticity of module detection

There were large difference between the number of tracked workers in the honey bees (mean ± sd: 938.2 ± 236.9 workers) and the ants (*L. niger* : 93.0 ± 35.2; *L. acervorum*: 64.1 ± 20.33; *T. nylanderi*: 114.2 ± 36.7). Similarly, there were large differences between the number of sites recorded in the honey bees (mean ± sd: 40702 ± 471 sites) and the ants (*L. niger* : 5367 ± 158; *L. acervorum*: 3121 ± 79; *T. nylanderi*: 6752 ± 334).

It is therefore possible that the inter-specific differences in degree of social and spatial compartmentation reported in the main text could be an artefact of our module detection method: for example, the stochasticity of the module detection algorithm may be stronger in smaller networks, resulting in less consistent identification of network modules and therefore lower apparent social and spatial compartmentation.

To evaluate the extent to which network size may affect the performance of the module detection algorithm, we conducted an additional analysis in which we randomly sub-sampled colonies to a comparably low number of

individuals, and used spatial grids with a comparably low number of sites. To do so, in each colony we randomly selected a subset of 6.8% (*A. mellifera*), 67.7% (*L. niger*) or 55.2% (*T. nylanderi*) of all tracked workers (*L. acervorum* colonies were not subsetted, as they had the smaller colony size of all species). This led to subsetted colony sizes of 63.5 ± 16.4, 63.65 ± 24, and 63.5 ± 20.3 workers in *A. mellifera*, *L. niger* and *T. nylanderi*. To ensure that we had sampled a representative subset of workers, we checked that the mean module scores of the sampled workers were each within 10% of that of the entire colony; if that was not the case, the subset was discarded and a new one was selected randomly. Furthermore, in *Apis*, *Lasius* and *Temnothorax*, we defined new hexagonal lattices with wider bins so the total number of sites would be as close to the number of sites consituting the *Leptothorax* grid as possible (*A. mellifera*: 3180 ± 15 ; *L. niger*: 3086 ± 20; *T. nylanderi*: 3053 ± 46). We then applied our entire analytical pipeline to these subsampled colonies and modified grids (Fig. S8).

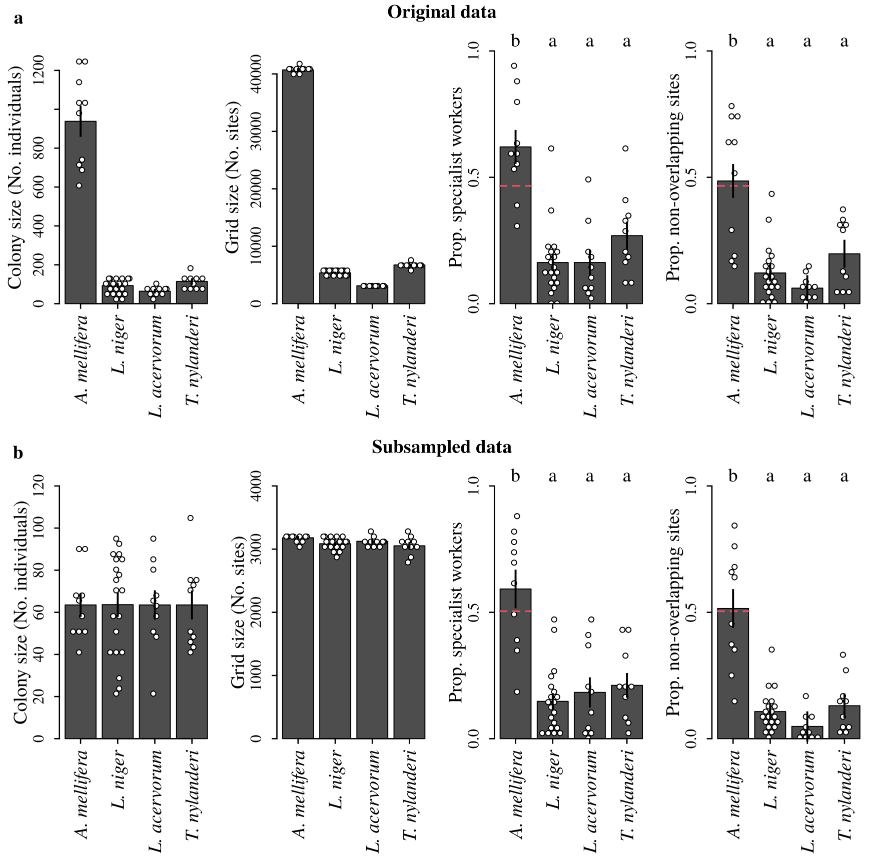

Figure S8: **Colony sub-sampling shows that inter-specific differences in social and spatial compartmentation are not an artefact of size-sensitive analysis methods.** Points, bars & whiskers represent colony values, cross-colony means & standard errors, respectively. Letters above bars indicate Benjamini-Hochberg corrected post-hoc contrasts (same letters: $p > 0.05$; different letters: $p < 0.05$) between all species pairs in general linear models (GLMs). The red lines show the means for *A. mellifera* when the two nurse modules were treated separately, rather than being pooled. All analyses based on n = 50 colonies (a) or n = 50 colony subsets (b). Source data are provided as a Source Data file.

If the inter-specific differences reported in the main text were an artefact due to the sensitivity of the module-detection algorithm to network size, then we would expect these differences to disappear when comparing the subsampled colonies. By contrast, if the inter-specific differences reported in the main text reflected real differences in the behaviour of honey bee vs ant workers, then they should still be present when comparing the subsampled colonies. Our results show that subsampling the number of sites and individuals to similar low numbers across all species did not erase the inter-specific differences detected over the full networks, indicating that they represent meaningful differences in behaviour between species and instead of being an artefact from our module detection method (Fig. S8).

Supplementary Note 9: Mixing between worker populations from different modules

The module scores of all sites and individuals were used to calculate the diversity (an entropy-based measure of heterogeneity) of visited sites for each individual, and the diversity of visitors for each site (see main text for detail). Fig. S9 represents the visitor diversity maps for all colonies in the experiment.

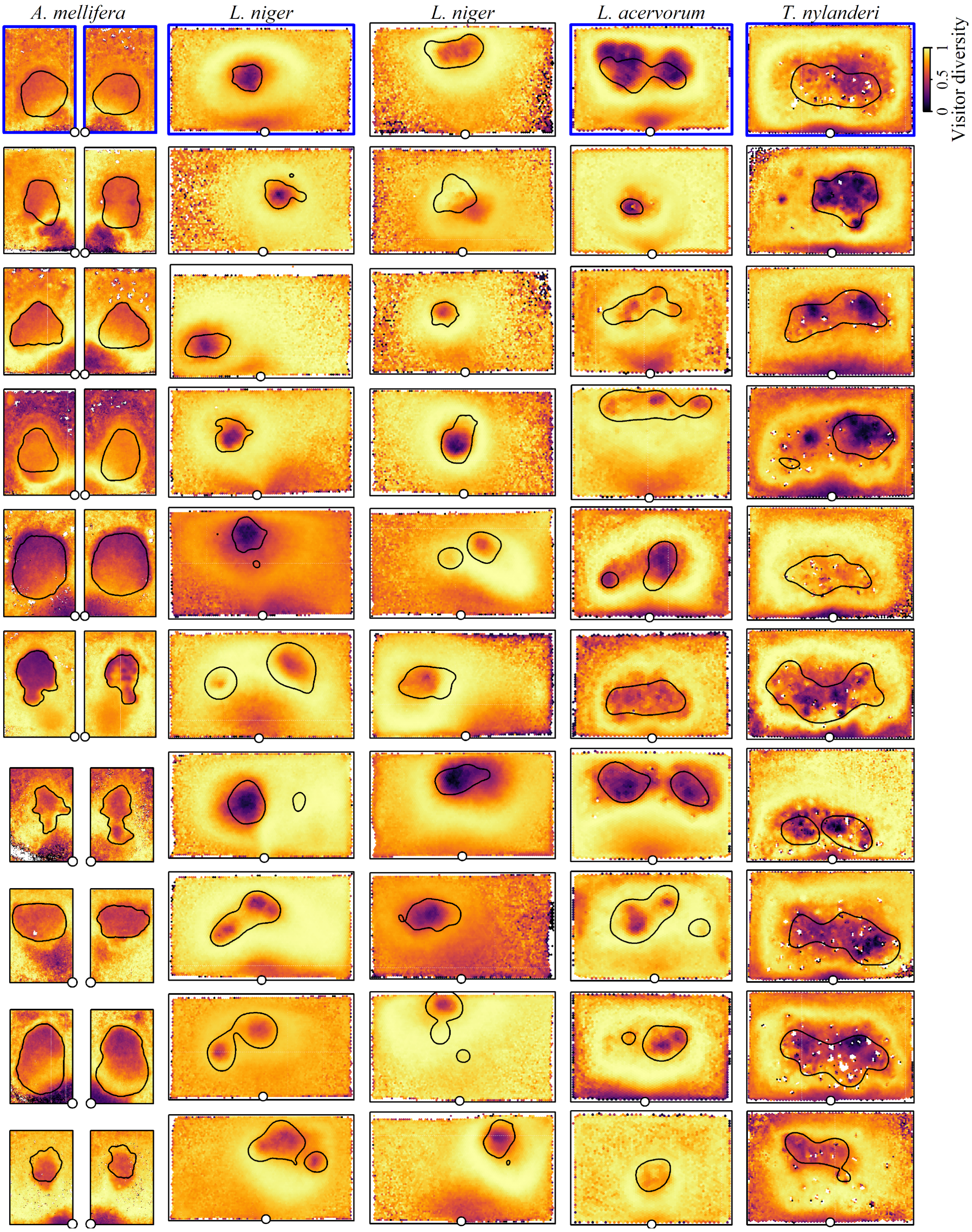

Figure S9: **Visitor diversity maps.** Sites are coloured according to the diversity index, D_s. The diversity index ranges from 0 (dark) to 1 (light). Sites with low visitor diversity are frequented by 'specialist' individuals – those that score highly for a single module. Sites with high visitor diversity are frequented by generalists and/or by an equal mix of specialists from different modules. The four colonies framed in blue in the top row correspond to the colonies used as an illustration in the main text.

Supplementary Note 10: Between-species similarities in spatial and social organisation

In order to establish whether the module score diversity (i) of site visitors, (ii) of visited sites and (iii) of contact partners differed between modules, we fitted general linear mixed models (LME) for each measure of diversity (Table S4). Models were fitted separately for the ants and the bees because they differed in the identity of their modules; thus 6 models were fitted in total. In all models the main effects were colony size and primary module. Colony identity was used as a random effect in all models. As the model for the ants encompassed three species, in this model we included species as a second random effect. Where necessary, the response variable was squared in order to achieve normality of residuals (residual skewness between -2.1 and 2.1 and kurtosis lower than 7.1). The effect of colony size was signigicant only in one of six models (effect of primary module on visited site diversity in ants; χ^2 = 5.8, d.f. = 1, p=0.02). In that model increasing colony size was associated with decreased visited site diversity (i.e. increased overall spatial compartmentation); though as mentioned above it is difficult to disentangle the effects of colony size *per se* from those of possible species-specific idiosyncrasies. The effect of the primary module was highly significant in all six models and the associated post-hoc tests are reported in (Table S4).

		Sites		***Individuals***			
		**Visitor diversity** ($D_{visitor,s}$)		**Visited site diversity** ($D_{visited,i}$)		**Contact partner diversity** ($D_{contacted,i}$)	
	**Contrast**	**z**	**p**	**z**	**p**	**z**	**p**
	Peripheral - Nurse	160.0	<0.0001	40.6	<0.0001	38.2	<0.0001
**Bees**	Forager - Nurse	138.7	<0.0001	22.1	<0.0001	54.2	<0.0001
	Forager - Peripheral	-13.2	<0.0001	-18.8	<0.0001	8.4	<0.0001
	Intermediate - Nurse	191.9	<0.0001	20.9	<0.0001	19.8	<0.0001
	Peripheral - Nurse	164.6	<0.0001	22.3	<0.0001	20.7	<0.0001
**Ants**	Forager - Nurse	35.3	<0.0001	0.64	0.52	6.6	<0.0001
	Peripheral - Intermediate	-64.1	<0.0001	-0.84	0.48	-1.2	0.24
	Forager - Intermediate	-173.8	<0.0001	-20.0	<0.0001	-14.9	<0.0001
	Forager - Peripheral	-144.2	<0.0001	-21.2	<0.0001	-15.1	<0.0001

Table S4: **The intermediate and peripheral modules have higher diversity indices than the nurse and forager modules.** Results from pairwise post-hoc tests in which p-values were corrected for multiple testing using the Benjamini-Hochberg method (LMEs with colony and ant species as random effects, main effect of primary module on visitor diversity (sites), honey bees: χ^2 = 30121, d.f. = 2, p<0.0001; ants: χ^2 = 58279, d.f. = 3, p<0.0001; main effect of primary module on visited site diversity (individuals), honey bees: χ^2 = 1764.0, d.f. = 2, p<0.0001; ants: χ^2 = 895.8, d.f. = 3, p<0.0001; main effect of primary module on contact partner diversity (individuals), honey bees: χ^2 = 3497.2, d.f. = 2, p<0.0001; ants: χ^2 = 680.2, d.f. = 3, p<0.0001). All analyses based on n = 12 494 workers or n = 613 100 sites drawn from n = 50 colonies.

We next tested whether the diversity of visitors varied depending on salient nest features (Table S5).

		**Predictor**							
		**Near nest entrance**				**On brood**			
**Response**	**Species**	β	χ^2	**df**	**p**	β	χ^2	**df**	**p**
	A. mellifera	-0.30	12511.9	1	<0.0001	-0.17	42905.5	1	<0.0001
Visitor diversity	*L. niger*	-0.17	3445.0	1	<0.0001	-0.22	10037.5	1	<0.0001
($D_{visitor,s}$)	*L. acervorum*	-0.24	1354.3	1	<0.0001	-0.26	4829.2	1	<0.0001
	T. nylanderi	-0.30	4159.9	1	<0.0001	-0.33	18966.5	1	<0.0001

Table S5: **Visitor diversity is lower on brood pile and near the nest entrance.** One LME model was fitted for each species using visitor diversity as response. For each model, we used one random effect (colony identity), and three main effects (on vs. away from brood, near vs. away from nest entrance, and colony size). The effect of colony size was non significant in all models (χ^2 < 0.9, d.f. = 1, p >0.34). The significance of other effects is reported in the table. In this table, the distance threshold used to distinguish between near and away from the nest entrance was set at 10% of the nest's diagonal length, but results were qualitatively similar and statistically significant for thresholds ranging from 2% to 25% of the nest's diagonal length. Note that the response variable was squared in order to achieve normality of residuals. All analyses based on n = 613 100 sites drawn from n = 50 colonies.

Supplementary Note 11: Describing movement patterns - focal-point attraction

The aim of this analysis was to determine the extent to which individual movement showed consistent directional biases towards their primary spatial module when they are outside that module. As the spatial modules produced by our method overlap and do not have clear edges, our first step was to identify boundaries delimiting each spatial module. This was done by building simplified nest maps (Fig. S10), in which each site was assigned to the majority primary module within that site's neighbourhood (i.e. the focal site and all 6 neighbouring sites).

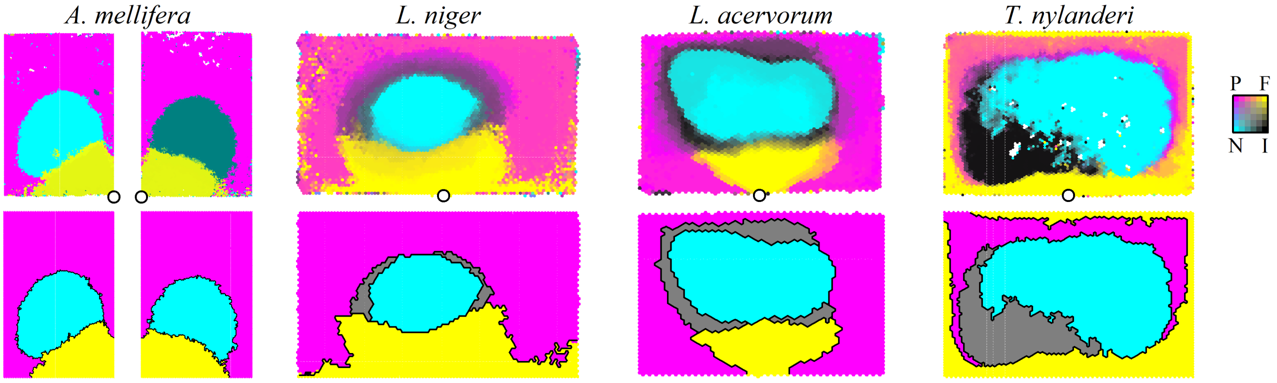

Figure S10: **Extracting module boundaries.** The top row shows the original spatial module maps for four colonies. The bottom row shows the corresponding simplified spatial module maps, with module boundaries overlaid in black.

We then calculated a taxis index for each individual i at each site s and toward each module M. To do so, we first calculated the mean resultant vector $\vec{v_{i,s}}$ of all trajectory steps starting at s when i was in the active state, where a trajectory step is defined as the segment connecting two successive fixes. Any step spanning more than 2 seconds (i.e. in which i was undetected for 4 or more successive frames) was excluded from the calculation to avoid including imprecise or incorrect direction values. The angle of the mean resultant vector corresponds the preferred (mean) direction of individual i at site s, while the length of the mean resultant vector represents the consistency in the direction of individual i at site s, ranging from 0 (no consistency) to 1 (all trajectory steps pointing in the exact same direction). The taxis $T_{M,i,s}$ of individual i at site s toward module M was then quantified as the projection of $\vec{v_{i,s}}$ on the vector $\vec{v_{M,s}}$ pointing from site s to the nearest point on the boundary of module M, i.e.:

$$T_{M,i,s} = ||\vec{v_{i,s}}||.cos(\theta_{M,i,s})$$

where $\theta_{M,i,s} \in \;]-\pi, +\pi]$ is the angular difference between $\vec{v_{i,s}}$ and $\vec{v_{M,s}}$. $T_{M,i,s}$ takes values ranging from -1 (individual always away from the module boundary) to 1 (individual always travels towards the module boundary). A negative taxis indicates repulsion from, whereas a positive taxis index indicates attraction towards, the module of interest (Fig. S11).

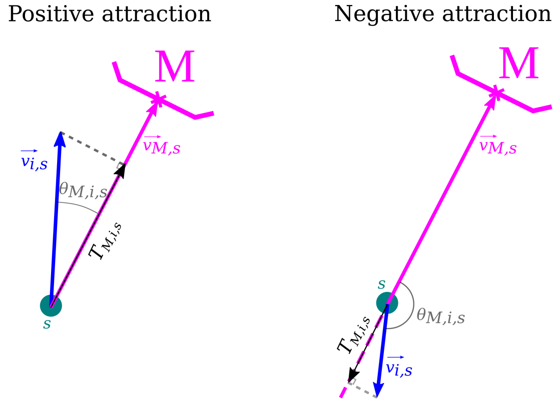

Figure S11: **Measuring attraction toward or away from module boundaries.** The green point indicates a focal site s. The solid magenta line shows the boundary of a focal module M, and the magenta arrow the vector $\vec{v_{M,s}}$ pointing from s to the nearest point on the boundary of M (magenta cross). The solid blue arrow the mean resultant vector $\vec{v_{i,s}}$ of all trajectory segments of a focal ant i starting at site s. The angle $\theta_{M,i,s}$ represents the angular difference between $\vec{v_{i,s}}$ and $\vec{v_{M,s}}$. The black arrow represents the taxis $T_{M,i,s}$ of individual i at site s toward module M, that is, the projection of $\vec{v_{i,s}}$ on $\vec{v_{M,s}}$. The left graph depicts a situation where $T_{M,i,s}$ is positive (attraction toward module M), whereas the right graph depicts a situation where $T_{M,i,s}$ is negative (repulsion from module M).

Supplementary Note 12: Describing movement pattern - locomotion adjustment

To quantify how individual locomotion depends concurrently on the module scores of the individual and that of the sites that it visits, we used linear mixed-effects (LME) modelling. Three mixed-effects models were run for each species. In the first mixed model, the response was the site-specific probability of being in the active state $P_{i,s}$ (Fig. S12 a-d). In the second, the response was the site-specific mean speed while in the active state (Fig. S12 e-h). In the third, the response was the site-specific mean absolute turn angle while in the active state (Fig. S12 i-l). All models included the following main effects: (i) the cosine similarity between the module scores of the individual i and site s, $\cos_{i,s}$, and (ii) colony size, i.e. the number of workers in the colony. Furthermore, all models shared the same random effects, namely, (i) colony identity, (ii) worker identity, (iii) site identity, and (iv) worker density, that is, the mean density of nestmates at a given site. To control for between-subjects and between-colony variation, individual identity was hierarchically nested within colony identity. Similarly, to control for between-sites variation in worker density, site identity was nested within worker density. The main coefficients and their significance are show in Fig. S12.

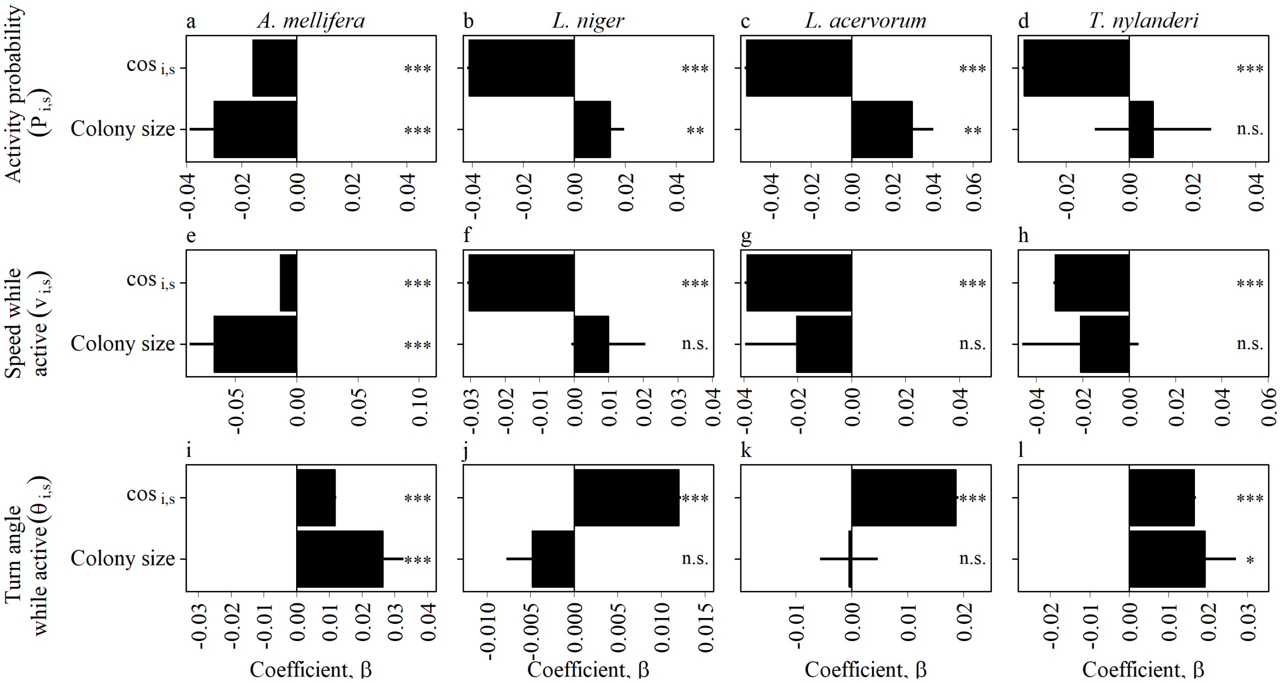

Figure S12: **Individual activity and movement are context-dependent.** Bars show the effect size (model coefficient, β) and standard error for the mixed effects models (LMEs) described in the text. Symbols indicate the significance of each effect (n.s.: $p > 0.05$; *: $p < 0.05$; **: $p < 0.01$; ***: $p < 0.001$) **(a-d)** Effect sizes for mixed models predicting the proportion of time an individual spent in the active state, $P_{i,s}$. **(e-h)** Effect sizes for mixed models predicting the site-specific mean speed for individuals that were in the active state. **(i-l)** Effect sizes for mixed models predicting the site-specific mean turn angle for individuals that were in the active state. Means were calculated over the absolute (unsigned) turn angles. Analyses based on n = 12 494 workers and n = 613 100 sites drawn from n = 50 colonies.

Supplementary Note 13: Agent-based simulation modelling

To test whether the individual-level rules identified in our analyses are sufficient to explain the maintenance of spatial segregation in social insects, we developed an agent-based simulation model for individual movement. In this model, agents were allowed to move within a two-dimensional area with periodic boundary conditions representing the nest. This area was divided into a hexagonal grid with a cell width of 1 (arbitrary units). The size of each agent was set to 4 units, so the cell width:body length ratio was the same as in our main analysis (1:4). Each simulation modelled the trajectory of a single agent as a stochastic process based on a correlated random walk (CRW). Several model variants representing alternative segregation rules were implemented by incorporating additional movement rules to the basic CRW model.

As our experiment involved four different study species, all simulations were repeated with different parameter values calibrated using tracking data from each species. For each model variant and each species, simulations were repeated to produce a number of independent trajectories comparable to our experimental colony sizes. Furthermore, for each species the dimensions of the nest were chosen so that the hexagonal grid contained approximately as many cells as in our experiment, and the number of simulated agents was equal to the mean colony size in our experiment (see Table S6; for simplicity in the honeybees we considered one side of the comb only). Sensitivity analyses with different nest dimensions and number of agents were additionally run to explore the extent to which inter-species differences may due to nest size and/or worker density (see detail below).

Correlated random walk (CRW) model

In each simulation, the agent was assigned a random initial activity state (active or inactive) and a random starting location within the nest. The first movement step was determined by randomly drawing a movement direction from a uniform distribution ranging from $-\pi$ to $+\pi$, and a movement distance from an exponential distribution with a rate depending on the activity state (see below). After this initialisation, all subsequent movements were determined using the following algorithm:

(i) Determine new activity state. Agents switched activity states stochastically with rates $P_{I \to A}$ (probability of switching from inactive to active) and $P_{A \to I}$ (probability of switching from active to inactive).

(ii) Determine movement distance. A movement distance was randomly drawn from an exponential distribution with rate R_I (inactive agent) or R_A (active agent); the larger the rate, the lower the mean distance.

(iii) Determine turn angle. A turn angle was randomly drawn from a wrapped normal distribution centered around 0 and wrapped between $-\pi$ and $+\pi$, with concentration parameter ρ_I (inactive agent) or ρ_A (active agent); the larger the concentration parameter, the lower the mean absolute turn angle.

(iv) Calculate new location. Movement distance and turn angle were then used to calculate the new coordinates. According to the periodic boundary conditions, if the new coordinates exceeded the boundaries of the square nest, the agent reappeared on the other side.

All parameters were parameterised using tracking data from each species (see Table S6).

Modulatory movement rules

In this section, we describe three sets of modulatory movement rules which were used – alone or in combination – to adjust the basic CRW model to introduce spatial segregation among agents. Importantly, the aim of these simulations was not to explore potential rules explaining the *emergence* of spatial segregation from an initially homogeneous environment, but to test whether our candidate modulatory movement rules were sufficient to explain the *maintenance* of spatial segregation in an already heterogeneous environment. Accordingly, at the beginning of simulations half of the agents were assumed to be 'central' (with a preferred spatial zone within the center of the nest) and the other half were assumed to be 'peripheral' (with a preferred spatial zone at the periphery of the nest).

(i) Focal-point attraction

Following Crall et al., 2018, to model attraction to/repulsion from the centre, at each time step we first determined a stochastic baseline movement vector $\overrightarrow{v_{CRW}}$ using the CRW model. If the agent was outside of its preferred zone (distance score DS superior to 0.5 for central agents or inferior to 0.5 for peripheral agents, see section (ii) below), we then modified this vector to incorporate long-range attraction to the home zone. To do so, we determined a 'bias vector' $\overrightarrow{v_{bias}}$ pointing from the agent location towards (central agents) or away from (peripheral agents) the nest centre. We then calculated a weighted sum between the CRW vector and the bias vector: $\overrightarrow{v_{weighted}} = (\mu \times \overrightarrow{v_{bias}} + (1 - \mu) \times \overrightarrow{v_{CRW}})$, where μ represented the strength of the directional bias and depended on the agent's activity state. We used a directional bias strength $\mu_I = 0$ when the agent was in the inactive state (pure correlated random walk), and $\mu_A = 0.01$ when the agent was in the active state (small directional bias; see Table S6). Finally, we adjusted the magnitude of this weighted vector so the resulting motion vector length was equal to the 'distance moved' determined by the CRW model: $\overrightarrow{v_{motion}} = \overrightarrow{v_{weighted}} \times \frac{\|\overrightarrow{v_{CRW}}\|}{\|\overrightarrow{v_{weighted}}\|}$.

	A. mellifera (single comb side)	*L. niger*	*L. acervorum*	*T. nylanderi*
**Experiment**				
Number of sites	20351 ± 235	5367 ± 158	3121 ± 79	6752 ± 334
Number of tracked individuals	469 ± 118	93 ± 35	64 ± 20	114 ± 37
**Simulation parameters**				
Nest side dimension	131.5	67.5	51.5	75.5
Number of cells	20064	5304	3120	6688
Number of agents	470	94	64	114
Worker density	0.023	0.018	0.021	0.017
Cell width	1	1	1	1
Agent body length	4	4	4	4
Default CRW model				
$P_{I\to A}$	0.00667	0.00223	0.00278	0.00167
$P_{A\to I}$	0.000596	0.000766	0.00122	0.000478
R_I	6.7	14.9	9.08	10.3
R_A	2.05	1.14	1.66	2.25
ρ_I	0.144	0.429	0.448	0.645
ρ_A	0.269	0.463	0.683	0.757
Focal-point attraction				
μ_I	0	0	0	0
μ_A	0.01	0.01	0.01	0.01
Local locomotion adjustment				
Θ	52.46	26.93	20.55	30.12
k	21	7.5	5	10.5
$P_{I\to A,Home}$	0.00636	0.00224	0.00214	0.00109
$P_{A\to I,Home}$	0.000672	0.00186	0.00241	0.000677
$R_{A,Home}$	2.31	1.53	2.39	2.98
$\rho_{A,Home}$	0.240	0.431	0.643	0.737
$P_{I\to A,Away}$	0.00707	0.00226	0.00316	0.00177
$P_{A\to I,Away}$	0.000537	0.000496	0.000867	0.000336
$R_{A,Away}$	1.89	1.01	1.44	2.02
$\rho_{A,Away}$	0.291	0.470	0.701	0.768
Boundary effect				
σ	0.120	0.154	0.111	0.0905

Table S6: **Simulation parameters.** Simulations were ran using 4 separate sets of parameters calibrated using tracking data from each of our study species. The top section of the table ('Experiment') shows the mean ± standard deviation of the number of sites and number of tracked individuals for each species in the main experiment; note that for the honeybees, these values were divided by two to reflect a single side of the comb. The bottom section of the table ('Simulation parameters') shows the parameters used in the main series of simultions for each modulatory movement rule. $P_{I\to A}$, $P_{A\to I}$ and their derivatives are expressed in $step^{-1}$ where one time step corresponds to 0.5 seconds (to match our experiment where tracking was performed at a rate of 2 frames per second); R_I, R_A and their derivatives are expressioned in $step.cell^{-1}$ where one cell width corresponds to 1/4 of the agent's body length; ρ_I, ρ_A, μ_I, μ_A, k and their derivatives are unitless; Nest side dimension, Agent body length and Θ is expressed in *cells*; σ is expressed in *radians.cell*.

(ii) Local locomotion adjustment

To model locomotion adjustment, we adjusted the probabilities of switching activity states ($P_{I\to A}$ and $P_{A\to I}$), the speed while active (rate parameter R_A) and the turn angle while active concentration parameter ρ_A) depending on location. To reflect our main findings, central agents had (i) a lower probability of being active (lower $P_{I\to A}$, higher $P_{A\to I}$), (ii) a lower speed while active (higher R_A), and (iii) a higher turn angle when active (lower ρ_A) at the centre of the nest (their primary module) than at the periphery. By contrast, peripheral ants had (i) a higher probability of being active (higher $P_{I\to A}$, lower $P_{A\to I}$), (ii) a higher speed while active (lower R_A), and (iii) a lower turn angle when active (higher ρ_A) at the centre than at the periphery of the nest (their primary module).

To implement these location-dependent parameter adjustments, each location was assigned a distance score

DS ranging from 0 (centre) to 1 (periphery) according to the following sigmoid function:

$$DS = \frac{d^k}{d^k + \Theta^k}$$

where d is the distance between the focal location and the centre of the nest, Θ is the distance of transition between the central and peripheral zones (value of d for which DS=0.5), and k the steepness of the function. In order for the central and peripheral zones to cover the same area, Θ was assigned the value

$$\Theta = \frac{nest\_side}{\sqrt{2\pi}}$$

where $nest\_side$ is the dimension of the square nest area used in the simulations. In the main series of simulations, to reflect the fact that our four model species have different transition zone steepnesses, the k parameter was chosen such that the proportion of grid cells with a DS of either 0 or 1 (after rounding to two decimal places) was the same as in the experiments (see Table S6); further sensitivity analyses with different steepness values were also run (see detail below).

At each time step, before applying the basal CRW algorithm, a local value Ψ_{local} was calculated for each parameter of interest Ψ ($P_{I \to A}$, $P_{A \to I}$, R_A or ρ_A) according to the following formula:

$$\Psi_{local} = \begin{cases} \Psi_{Home} + DS * (\Psi_{Away} - \Psi_{Home}), & \text{if agent is central} \\ \Psi_{Home} + (1 - DS) * (\Psi_{Away} - \Psi_{Home}), & \text{if agent is peripheral} \end{cases}$$

where Ψ_{Home} and Ψ_{Away} represented the values of the parameter of interest in the primary module ('at home') or away from the primary module, respectively. Ψ_{Home} and Ψ_{Away} were parameterised for each species using tracking data from individual-site pairs with a cosine-similarity of 1 (Ψ_{Home}) or 0 (Ψ_{Away}; see Table S6).

(iii) Boundary effect

To model changes in worker direction in zones of transition between spatial modules, we first calculated the gradient field $|g|_{DS}$ of the rounded DS scores in the same way as we calculated the gradient field of module scores in the main analysis (see Methods in the main text). As DS scores increased from 0 in the centre to 1 at the periphery, peripheral agents were considered to travel 'up-gradient' of their primary module when they travelled upwards in the DS score gradient field, whereas central agents were considered to travel 'up-gradient' of their primary module when they travelled downwards in the DS score gradient field. Conversely, peripheral agents were considered to travel 'down-gradient' of their primary module when they travelled downwards in the DS score gradient field, whereas central agents were considered to travel 'down-gradient' of their primary module when they travelled upwards in the DS score gradient field. At each time step, we first determined a stochastic baseline movement vector $\overrightarrow{v_{CRW}}$ using the CRW model, characterised by a step length L_{CRW} and a turn angle θ_{CRW}. We then calculated an adjustment factor Σ_s as follows:

$$\Sigma_s = \sigma * |g|_{DS,s}$$

, where s is the site where the agent was at the beginning of the time step, σ is the turn angle adjustment factor, and $|g|_{DS,s}$ is the field steepness of DS scores at site s. We then computed a resulting turn angle $\theta_{boundary}$ according to the following formula:

$$\theta_{boundary} = \begin{cases} \theta_{CRW} + \Sigma_s, & \text{if the agent was travelling 'down-gradient' of its module at the previous time step} \\ \theta_{CRW} - \Sigma_s, & \text{if the agent was travelling 'up-gradient' of its module at the previous time step} \end{cases}$$

The resulting movement vector was characterised by step length L_{CRW} and turn angle $\theta_{boundary}$. Thus, as in our main analysis, agents were more likely to turn sharply if they were traveling away from their primary module and less likely to turn sharply if they were traveling towards their primary module, and these effects were stronger in transition zones (steeper gradients). Parameter σ was calibrated for each species using the empirical tracking data (see Table S6).

Model predictions on individual movement metrics

Our experimental data provided support for the *locomotion adjustment* and the *boundary effect* rules in all species (see main manuscript). To validate these findings, it is important to demonstrate that they are not due to spurious correlations between our input variable (number of visits made by each individual to each site) and our output analysis variables (proportion of time active, speed while active and turn angle while active, and direction-dependent modulation of turning behaviour for individuals at the boundary of their primary module), which would indicate circularity. Furthermore, it is also important to establish whether the same movement patterns which we identified in our analysis of empirical data could have been a byproduct of more than one of our candidate movement mechanisms.

To that end, we analysed the synthetic trajectories produced by simulations in which each of our three candidate mechanisms was implemented in isolation (F.P.A.: *focal-point attraction*; B.E.: *boundary effect*; L.A.: *locomotion adjustment*), or both local mechanisms were combined (B.E. + L.A.: *boundary effect + locomotion adjustment*). All simulations were run for a total of 10^7 time steps to ensure that any inherent trends between the variables of interest had time to emerge. Next, for the four types of synthetic trajectories we performed the same analyses that as described in the main text for the empirical trajectories, namely, (i) the taxis index difference between resident and non-resident agents for each focal zone (centre or periphery) at each site ; (ii) the proportion of time spent in the active state by each agent at each site; (iii) the mean speed of each agent at each site while in the active state; (iv) the mean absolute turn angle of each agent at each site while in the active state; and (v) the relative turn angle between resident and non-resident agents when travelling 'up-gradient' or 'down-gradient' of each focal module. We then tested whether the same trends emerged in the synthetic trajectories as in the observed data.

Predictions of taxis indices, and how they compare with the empirical trends, are shown in Fig. S13. Only the synthetic trajectories produced by the model involving the *focal-point attraction* mechanism (F.P.A.) exhibit consistent long-range attraction beyond two body lengths from the focal module border.

Predictions of movement characteristics associated with diffusivity, and how they compare with the empirical trends, are shown in Fig. S14. Only the synthetic trajectories produced by the two models involving the *locomotion adjustment* mechanism (L.A. and B.E. + L.A.) exhibit location-dependent modulation of movement diffusivity, in agreement with the empirical data.

Finally, predictions of relative turn angle for simulated agents visiting sites in the transition zone between the central and peripheral 'modules', and how they compare with the empirical trends, are shown in Fig. S15. Only the synthetic trajectories produced by the two models involving the *boundary effect* mechanism (B.E. and B.E. + L.A.) exhibit direction- and steepness-dependent modulation of turning behaviour, in agreement with the empirical data.

Overall, these analysis show that the predictions of the three candidate mechanisms do not overlap, i.e., only the *focal-point attraction* mechanism predicts long-range attraction of individuals towards their primary module (Fig. S13), only the *locomotion adjustment* model predicts location-dependent changes in movement diffusivity (Fig. S14), and only the *boundary effect* model predicted that turning behaviour of workers approaching their primary module boundary depends upon the approach direction and the gradient steepness (Fig. S15).

This is important, because if our main findings were due to spurious circular correlations whereby the individual movement patterns highlighted in our empirical analysis systematically arise from a heterogeneous distribution in site-visit patterns between group of workers regardless of the actual underlying mechanisms, then we would have expected all models to produce (i) a negative association between proportion of time active and agent-site similarity, (ii) a negative association between speed while active and agent-site similarity, (iii) a positive association between turn angle while active and agent-site similarity, and (iv) a direction-dependent modulation of turning behaviour for individuals at the boundary of their primary module. As this was not the case, we can confidently exclude the suggestion that our findings are due to spurious circular correlations.

Furthermore, across all movement metrics considered and all species, the local model combining both local mechanisms (*boundary effect* and *locomotion adjustment*) closely matched the empirical data, both in terms of statistical associations and in the order of magnitude of effect sizes (Fig. S13-S15). The only exception was long-range attraction in the honeybee, which was detected in the empirical data but not in the local model (Fig. S13). Overall, our simulations results are therefore consistent with the *boundary effect* and *locomotion adjustment* mechanisms acting as general organising rules underlying spatial organisation in social insect nests, with the honeybees additionally displaying long-range attraction to their primary module.

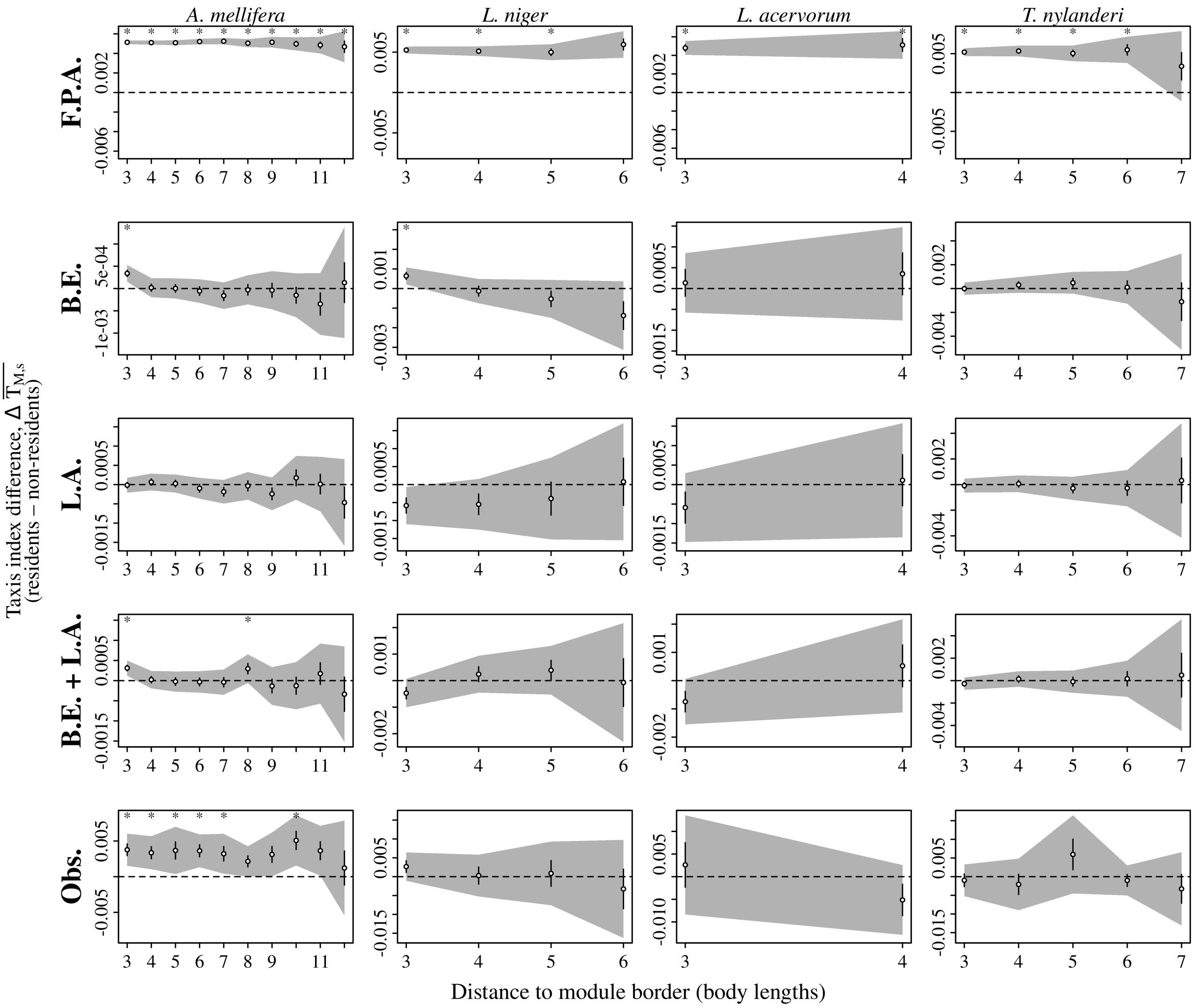

Figure S13: **Quantifying redundancy between model predictions I - long range attraction.** Points indicate the mean signed difference in the taxis indices of resident versus non-resident workers as a function of distance to the focal module border. Vertical bars indicate standard errors, and shaded areas represent the 95% confidence intervals. Asterisks indicate distances at which there were statistically significant differences (after Benjamini-Hochberg corrections) between the taxis of residents and non-residents. Panels are organised by model (rows), and by species (columns). Besides the observed trajectories (Obs.) in the honeybee (*A. mellifera*), only the trajectories produced by the focal point attraction model (F.P.A.) exhibit consistent long-range attraction (i.e., significantly greater taxis index of residents vs. non-residents at distances greater than 2 body lengths from the focal module border, after Benjamini-Hochberg correction for multiple testing). Observed data: analyses based on n = 12 494 workers or n = 613 100 sites drawn from n = 50 colonies. Simulated data: analyses for each model based on n = 742 agents or n = 35 176 sites. Source data are provided as a Source Data file.

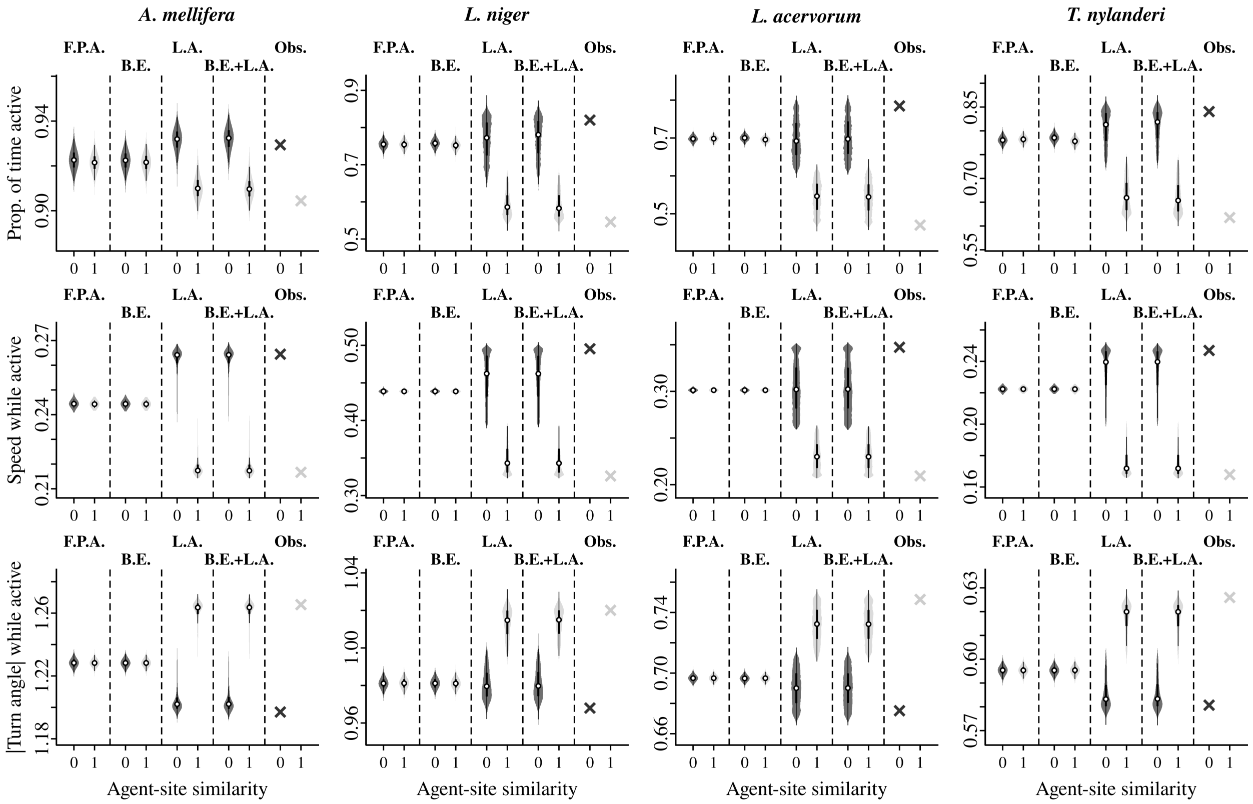

Figure S14: **Quantifying redundancy between model predictions II - movement diffusivity.** Panels show distributions of three metrics for measuring movement diffusivity (the proportion of time active, mean speed and turn angle while active), conditional upon the cosine similarity between the scores of the agent, and the site it visits (0 - completely dissimilar scores, 1 - identical scores). Light & dark-grey violin pairs indicate the predictions for a particular model, and for the empirical trajectories, as indicated by the abbreviations. Columns show the analyses for each species, as indicated by the headings. Location-dependent modulation of movement diffusivity occurs only in the observed trajectories (Obs.), and in the two models that incorporate locomotion adjustment (L.A. & L.A.+B.E.). Observed data: analyses based on n = 12 494 workers or n = 613 100 sites drawn from n = 50 colonies. Simulated data: analyses for each model based on n = 742 agents or n = 35 176 sites. Source data are provided as a Source Data file.

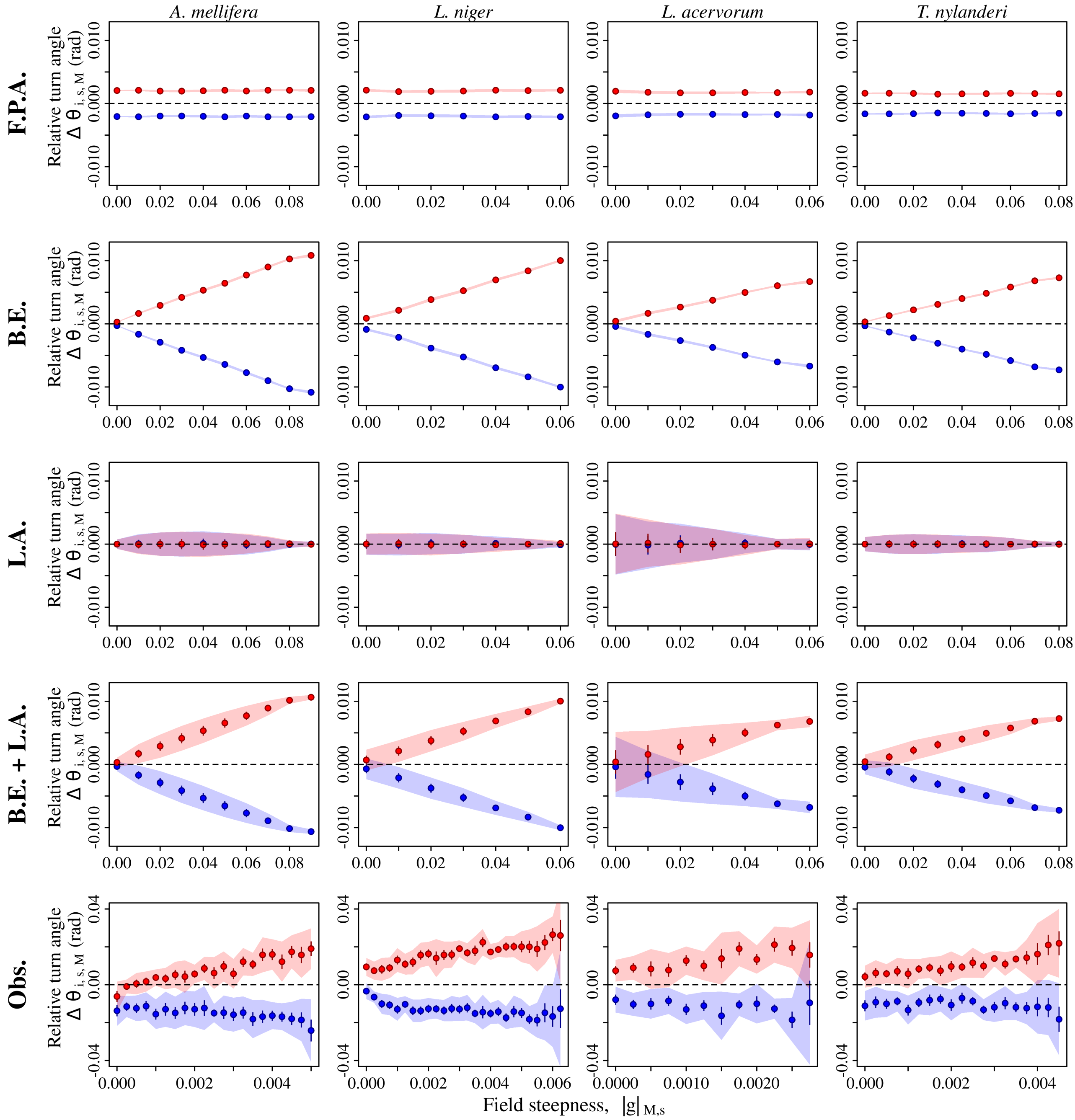

Figure S15: **Quantifying redundancy between model predictions III - turning behaviour.** Panels are organised by model (rows), and by species (columns). Besides the observed trajectories (Obs.), only the trajectories produced by models involving the *boundary effect* mechanism (B.E. & B.E.+L.A.) exhibit (i) a proportional increase in turning behaviour by residents approaching the module boundary from within (i.e., heading down-gradient - red points) as a function of increasing field steepness, and (ii) a proportional decrease in turning behaviour by residents approaching the boundary from outside (i.e., heading up-gradient, blue points) as a function of increasing field steepness. Observed data: analyses based on n = 12 494 workers or n = 613 100 sites drawn from n = 50 colonies. Simulated data: analyses for each model based on n = 742 agents or n = 35 176 sites. Source data are provided as a Source Data file.

Local model: species-specific parameters

To test whether the two universal rules highlighted in our main analysis (*locomotion adjustment* and *boundary effect*) were sufficient to explain the maintenance of spatial segregation in social insect nests, we implemented the following four model variants: (i) CRW; (ii) CRW + *boundary effect*; (iii) CRW + *locomotion adjustment*; (iv) CRW + *locomotion adjustment* + *boundary effect*. To be as close to our experimental conditions as possible, simulations were carried out for a total number of 518,400 time steps, which corresponds to the number of trajectory steps included in our main analysis (three day of continuous tracking at a rate of 2 frames per second). Fig. S16 shows the simulation outcome for all four models and four species.

We found that on its own, each of the two local rules (*locomotion adjustment* and *boundary effect*) was sufficient to induce some degree of spatial segregation between the two categories of agents (central and peripheral) compared to the baseline CRW model (Fig. S16a-b). However, the *locomotion adjustment* rule alone seemed more effective at inducing spatial segregation than the *boundary effect* rule alone, as it led to a greater increase in weighted modularity (Fig. S16b) and lower degree of spatial overlap between zones (Fig. S16a). Furthermore, the combination of the two local rules further reinforced spatial segregation between zones, with the highest values of weighted modularity and complete spatial segregation between modules (no overlapping site) in all species (Fig. S16a-b). Overall, our simulations confirmed that the two universal local rules highlighted in our analysis are sufficient to induce complete spatial segregation between modules, at least in the conditions used in our experiments.

Local model: sensitivity analysis on steepness and worker density

To explore the extent to which the degree of spatial segregation may depend on the steepness of the transition zone (model parameter k) and on worker density, we performed a sensitivity analysis in which the steepness parameter k and the worker density (number of simulated agents divided by number of grid cells) was systematically varied over a range of values encompassing all experimental values, whilst all other parameters were calibrated using species-specific tracking data (Fig. S17).

Increasing steepness led to increased spatial segregation between modules (higher proportion of non-overlapping sites and higher modularity) in all species and all model variants, whereas worker density in the nest had little to no effect on spatial segregation. In agreement with simulation results with species-specific parameters, the *locomotion adjustment* rule alone produced a greater degree of spatial segregation than the *boundary effect* rule alone through the entire range of parameters tested, and the combination of the two rules further reinforced spatial segregation. Surprisingly, although our experimental data revealed a greater degree of spatial segregation in the honey bees than in the ants, in our simulations the opposite was true: simulations with honeybee parameters typically resulted in much lower porportions of non-overlapping sites than simulations with ant parameters, and the combination of both local rules was necessary to obtain complete spatial segregation (i.e. no overlapping sites) between modules, whereas in the ants only one local rule was sufficient to induce complete spatial segregation over a broad range of parameter values. This apparent discrepancy between simulation results and observations could be explained by two non mutually exclusive factors: (i) the cues that honeybee respond to (e.g. content of the wax cells) may be much more clear-cut than the cues that the ants respond to (e.g. pheromone/hydrocarbon blend), resulting in much steeper transition between zones in honeybee than in ant nests, and (ii) spatial segregation in the honeybees may be further reinforced by longer-range global attraction to their primary module, as shown in Fig. S13.

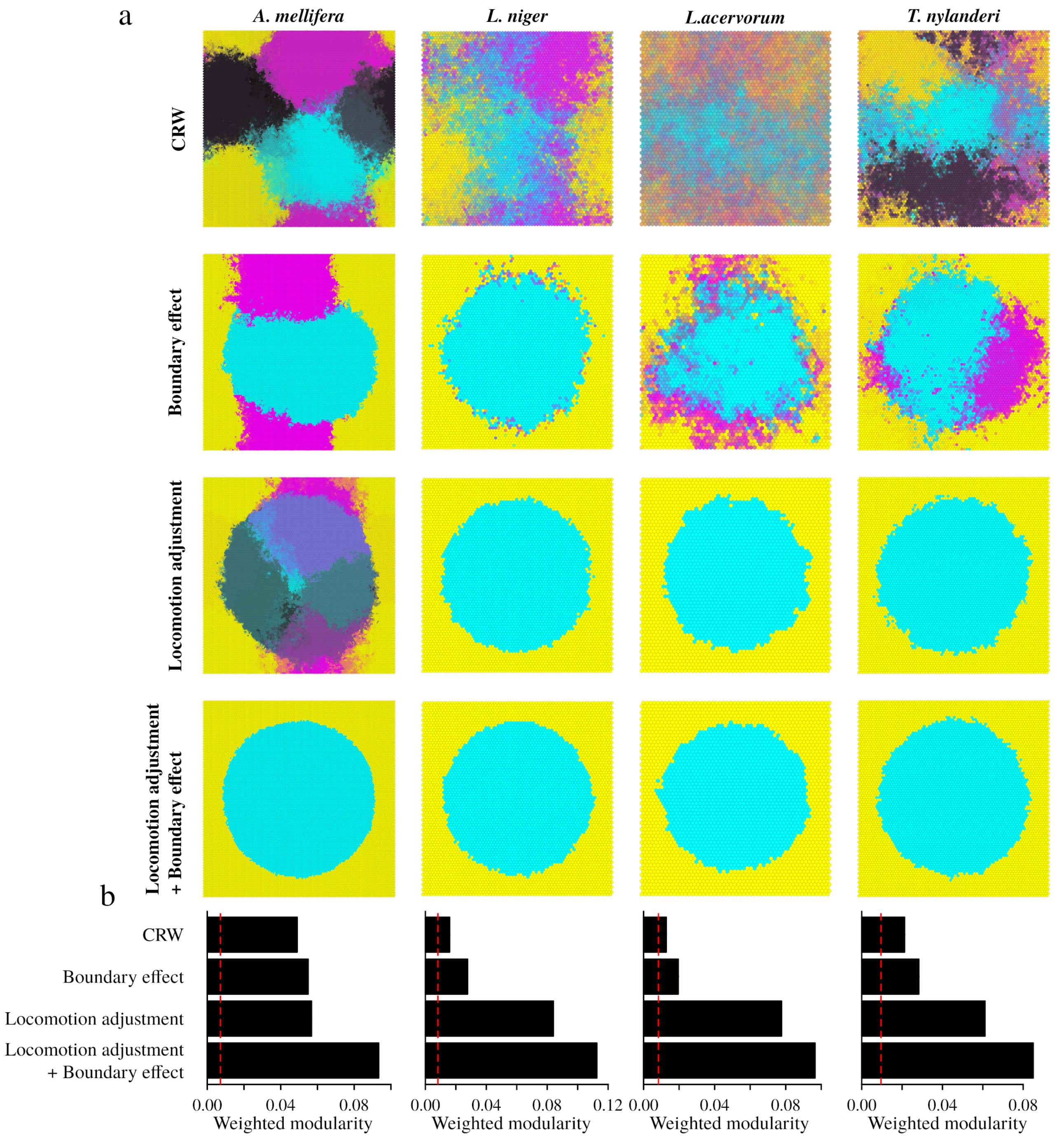

Figure S16: **Simulations using species-specific parameters: comparing alternative local models.** **(a)** Spatial module maps produced by running our main analysis algorithm on sets of simulated trajectories corresponding to four model variants (rows) and four sets of species-specific parameters (columns). Grid cells are coloured according to the linear (subtractive) combination of the colours for each detected module, weighted by its module scores (CMYK colour space); the most peripheral module is shown in yellow and the most central module in cyan. Additional detected modules are shown in magenta and black. **(b)** Bars and whiskers show the mean and standard error of the weighted modularity produced by repeated iterations of the module detection algorithm for each model variant (rows) and each species (columns); standard errors were so small that whiskers are barely visible to the naked eye. Dashed red lines indicate the mean weighted modularity for the corresponding permuted networks, as in Fig. S3. Analyses for each model based on n = 742 agents or n = 35 176 sites. Source data are provided as a Source Data file.

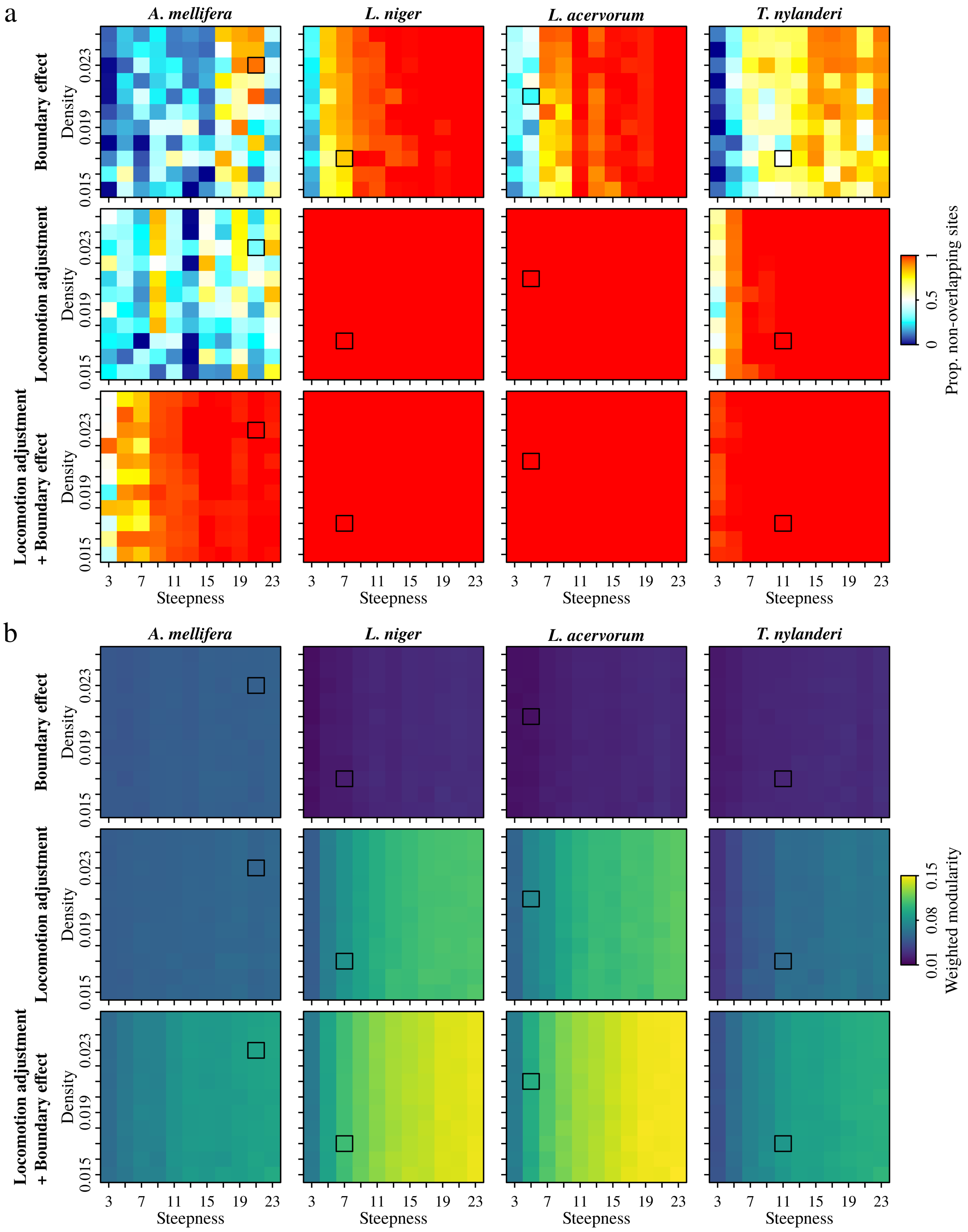

Figure S17: **Sensitivity analysis.** **(a)** Proportion of non-overlapping sites and **(b)** mean weighted modularity for each model variant and species , with systematically varied values of density and steepness. Analyses for each model and each pair of density and steepness values based on n = 742 agents or n = 35 176 sites. Source data are provided as a Source Data file.

Supplementary Note 14: Detecting physical contacts between workers

Physical contacts between pairs of individuals were identified using the method of Mersch et al. (2013) (Fig. S18).

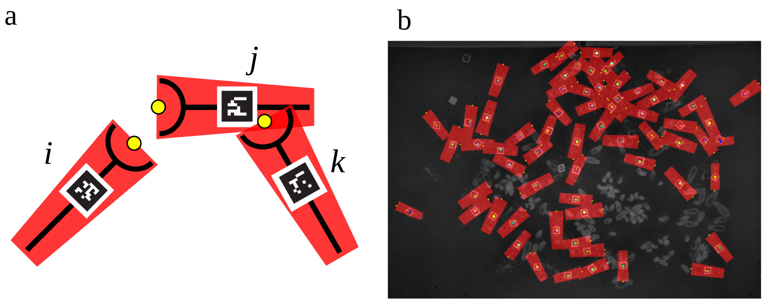

Figure S18: **Inferring physical contacts. (a)** Individual workers (black 'skeletons') are modelled as trapezoids centered on the tag. The narrow end of the trapezoid covers the tail of the individual, and the wider end covers the head and antennae. Workers are defined as being in physical contact when the 'interaction point' of either worker (yellow point) is inside the trapezoid of the other. Here, i is not in contact with j, but j is in contact with k. **(b)** Example tracking image from a *L. acervorum* colony. Trapezoids are overlaid upon each ant.

Supplementary Note 15: Identifying bouts of activity and inactivity

To classify trajectory segments according to whether a worker was in an active or inactive state, we used a three-step process in which trajectories were first segmented at 'change points', corresponding to conspicuous changes in movement. Trajectory segmentation was carried out using the *changepoint* package version 2.2.2 (Killick and Eckley, 2014) for *R*, which identified points at which the time-series of the instantaneous speed exhibited a regime change in both the mean and the variance (Fig. S19a, Barraquand and Benhamou 2008).

Next, each trajectory segment was characterized using seven statistics, namely (i) the mean, standard deviation and coefficient of variation of the motion speed, (ii) the mean, standard deviation and coefficient of variation of the sequential absolute turn angles, and (iii) the duration of the trajectory segment. These seven summary statistics for all trajectory segments were pooled across all individuals within each colony, and subjected to a principal components analysis. Scatterplots of the first and second principal components showed a characteristic L-shaped distribution for all species and all colonies (PCA, Fig. S19b), suggesting that there are indeed two classes of trajectory segments. We thus finally subjected the top 10 principal components of each PCA to a k-means cluster analysis, using k=2 (two groups). The resulting binary classification scheme allowed us to assign each trajectory segment to one of two categories: one consisting of fast and straight locomotion with high speed variation but low turn angle variation ('active' bouts), and another consisting of slow and tortuous locomotion, with low speed variation but higher turn angle variation, ('inactive' bouts; Fig. S19c).

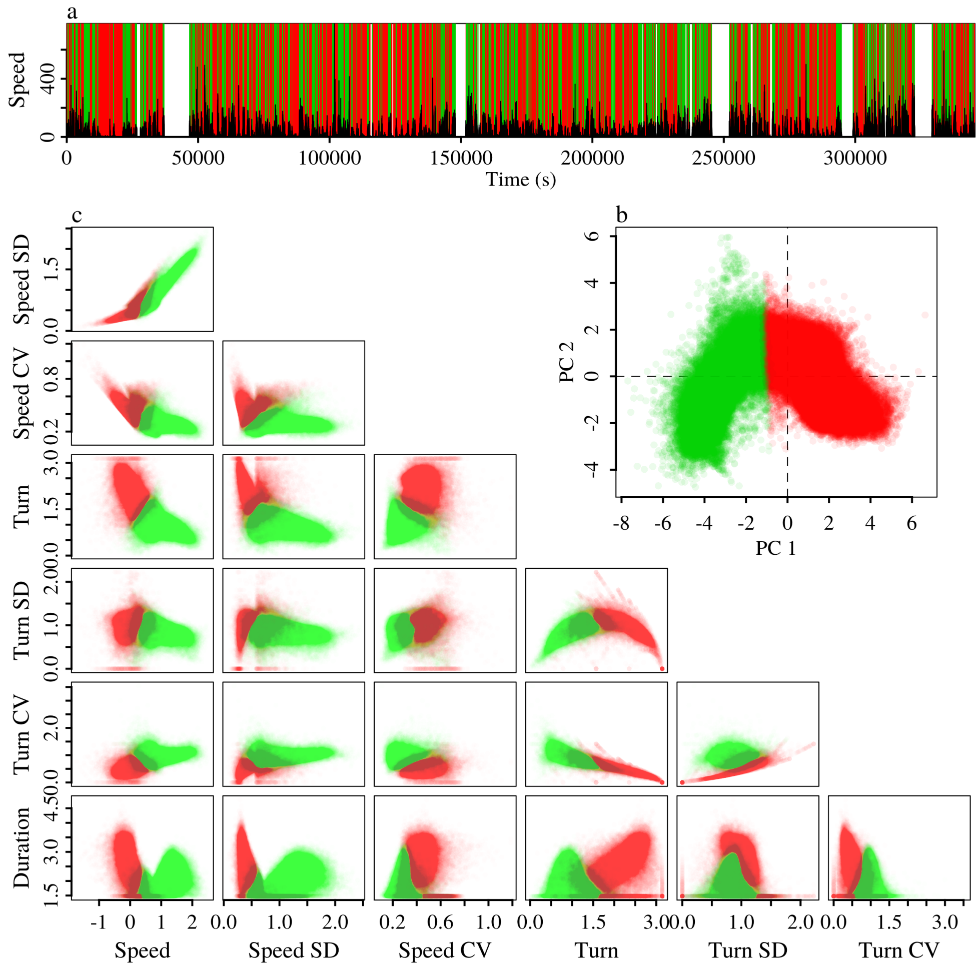

Figure S19: **Trajectory segments fall into two clusters. (a)** Three-day time series showing the speed of a single *T. nylanderi* worker (colony 10, ant 114). The coloured bars show the corresponding sequence of activity states produced by the combination of PCA, and k-means cluster analysis. Green (lighter shade): 'active' state; red (darker shade): 'inactive' state. White regions indicate times when the ant was not visible. **(b)** Principal components analysis (PCA) for all trajectory segments from all ants in colony colony 10. Each point represents a trajectory segment. Green (lighter shade) - trajectory segments classified as 'active' by k-means cluster analysis. Red (darker shade) - segments classified as 'inactive'. Prior to the PCA, the three speed variables were subjected to a log transformation, and all seven variables were centered and scaled. **(c)** Scatter plot matrix for the seven summary statistics used to characterize each trajectory segment. Each point represents a trajectory segment. Colours indicate activity state (green (lighter shade): 'active' state; red (darker shade): 'inactive' state).

Supplementary Note 16: Measuring site-specific activity and movement

In this section we detail the calculation of the three measures used to quantify the movement of a given individual i when visiting a given site s: the activity probability, the speed while active, and the turn angle while active.

All site-specific measures were based upon the trajectory of each individual (Fig. S20a), and the activity classifications (active vs. inactive) described above. The site-specific activity probability $P_{i,s}$ was defined as the time that individual i was in the active state when visiting site s, divided by the total time that it spent at that site (Fig. S20 c,b respectively). Thus, high a value indicates that i tended to be in the active state when visiting s, whereas low values indicate i was typically inactive at s.

To further charactertise movement properties *while individuals are in motion* and test how they vary across space, we also calculated the mean speed while active, $v_{i,s}$ (Fig. S20d) and the mean turn angle while active, $\theta_{i,s}$ (Fig. S20e) for each individual at each site. Both measures were calculated uniquely based on the trajectory segments in which the individual was in the *active* state; hence they were independent from (and uncorrelated with) the site-specific activity probability.

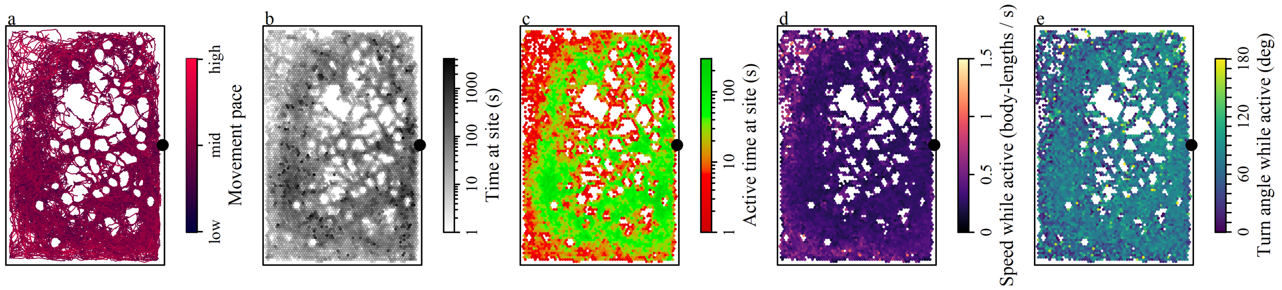

Figure S20: **Mapping spatial activity distributions. (a)** Trajectory for a single ant (*T. nylanderi*, colony 10, ant 114). Trajectory segment colour intensity indicates the speed of the ant (light red - fast, dark red - slow). The black point indicates the nest entrance. The empty 'voids' indicate the location of large brood items. **(b)** Spatial density estimation. Tile colours represent the total amount of time that the ant spent at a given site. **(c)** Spatial distribution of activity, $P_{i,s}$. Tile colours represent the summed time ant 114 was in the 'active' state whilst at a given site. **(d)** Site-specific mean speed while in the active state, $v_{i,s}$. **(e)** Site-specific mean turn angle while in the active state, $\theta_{i,s}$.

Supplementary References

Baracchi, D. and A. Cini (2014). A socio-spatial combined approach confirms a highly compartmentalised structure in honeybees. *Ethology 120*(12), 1167–1176.

Barraquand, F. and S. Benhamou (2008). Animal movements in heterogeneous landscapes: identifying profitable places and homogeneous movement bouts. *Ecology 89*(12), 3336–3348.

Beckett, S. J. (2016). Improved community detection in weighted bipartite networks. *Royal Society Open Science 3*(1), 140536.

Briscoe, A. D. and L. Chittka (2001). The evolution of color vision in insects. *Annu. Rev. Entomol. 46*(1), 471–510.

Connor, E. F. and D. Simberloff (1979). The assembly of species communities: Chance or competition? *Ecology 60*(6), 1132–1140.

Crall, J. D., C. M. Switzer, R. L. Oppenheimer, A. N. F. Versypt, B. Dey, A. Brown, M. Eyster, C. Guérin, N. E. Pierce, S. A. Combes, et al. (2018). Neonicotinoid exposure disrupts bumblebee nest behavior, social networks, and thermoregulation. *Science 362*(6415), 683–686.

Dormann, C. F. and R. Strauss (2014). A method for detecting modules in quantitative bipartite networks. *Methods in Ecology and Evolution 5*(1), 90–98.

Fiala, M. (2005, October). Comparing ARTag and ARToolkit Plus fiducial marker systems. In *HAVE 2005, IEEE International Workshop on Haptic Audio Visual Environments and Their Applications*, Ottawa, Canada.

Johnson, B. R. (2008). Global information sampling in the honey bee. *Naturwissenschaften 95*(6), 523–530.

Killick, R. and I. A. Eckley (2014). Changepoint: An R package for changepoint analysis. *Journal of Statistical Software 58*(3), 1–19.

Mersch, D. P., A. Crespi, and L. Keller (2013). Tracking individuals shows spatial fidelity is a key regulator of ant social organization. *Science 340*(6136), 1090–1093.

Olson, E. (2011, May). AprilTag: A robust and flexible visual fiducial system. In *Proceedings of the IEEE International Conference on Robotics and Automation (ICRA)*, pp. 3400–3407. IEEE.

Patefield, W. (1981). Algorithm AS 159: an efficient method of generating random R × C tables with given row and column totals. *Journal of the Royal Statistical Society. Series C (Applied Statistics) 30*(1), 91–97.

Poisot, T. (2013). An *a posteriori* measure of network modularity. *F1000Research 2*(130), 1–6.

R Core Team (2016). *R: A Language and Environment for Statistical Computing.* Vienna, Austria: R Foundation for Statistical Computing.

Seeley, T. D. (1982). Adaptive significance of the age polyethism schedule in honeybee colonies. *Behavioral Ecology and Sociobiology 11*(4), 287–293.
