# Supplementary material for: Two simple movement mechanisms for spatial division of labour in social insects
Source: Nat Commun. 2022 Nov 15;13:6985. doi: 10.1038/s41467-022-34706-7 (PMC9666475; doi:10.1038/s41467-022-34706-7)
Supplement: Supplementary file 2 — Description of Additional Supplementary Files [file 41467_2022_34706_MOESM2_ESM.pdf]

Description of Additional Supplementary Files

File Name: Supplementary Movie 1
Description: Application of the LargeVis dimensionality reduction algorithm to the high-dimensional worker task profiles. Workers belonging to different spatial modules exhibit different task profiles. Points represent different individuals. Point separation indicates task profile similarity. Point colours indicate the module score profiles. The module scores were not used in the LargeVis analysis or in the task definitions.
