# Supplementary material for: Two simple movement mechanisms for spatial division of labour in social insects
Source: Nat Commun. 2022 Nov 15;13:6985. doi: 10.1038/s41467-022-34706-7 (PMC9666475; doi:10.1038/s41467-022-34706-7)
Supplement: Supplementary file 4 — Reporting Summary [file 41467_2022_34706_MOESM4_ESM.pdf]

nature portfolio

Corresponding author(s): Thomas Owen Richardson

Last updated by author(s): Oct 27, 2022

Reporting Summary

Nature Portfolio wishes to improve the reproducibility of the work that we publish. This form provides structure for consistency and transparency in reporting. For further information on Nature Portfolio policies, see our Editorial Policies and the Editorial Policy Checklist.

Statistics

For all statistical analyses, confirm that the following items are present in the figure legend, table legend, main text, or Methods section.

n/a	Confirmed	
☐	☒	The exact sample size (*n*) for each experimental group/condition, given as a discrete number and unit of measurement
☐	☒	A statement on whether measurements were taken from distinct samples or whether the same sample was measured repeatedly
☐	☒	The statistical test(s) used AND whether they are one- or two-sided<br>*Only common tests should be described solely by name; describe more complex techniques in the Methods section.*
☐	☒	A description of all covariates tested
☐	☒	A description of any assumptions or corrections, such as tests of normality and adjustment for multiple comparisons
☐	☒	A full description of the statistical parameters including central tendency (e.g. means) or other basic estimates (e.g. regression coefficient) AND variation (e.g. standard deviation) or associated estimates of uncertainty (e.g. confidence intervals)
☐	☒	For null hypothesis testing, the test statistic (e.g. F, t, r) with confidence intervals, effect sizes, degrees of freedom and P value noted<br>*Give P values as exact values whenever suitable.*
☒	☐	For Bayesian analysis, information on the choice of priors and Markov chain Monte Carlo settings
☐	☒	For hierarchical and complex designs, identification of the appropriate level for tests and full reporting of outcomes
☒	☐	Estimates of effect sizes (e.g. Cohen's *d*, Pearson's *r*), indicating how they were calculated

Our web collection on statistics for biologists contains articles on many of the points above.

Software and code

Policy information about availability of computer code

Data collection	All data were collected using the protocols and software described in :<br>- Mersch, D. P., et.al. Tracking individuals shows spatial fidelity is a key regulator of ant social organization. Science 340, 1090 (2013).<br>- Stroeymeyt et al., Social network plasticity decreases disease transmission in a eusocial insect. Science 362(6417):941-945 (2018).<br>- Worker trajectories and physical contacts were extracted using freely available software; https://github.com/laurentkeller/anttrackingUNIL
Data analysis	The automated tracking datasets generated for the current study, and code for constructing the bipartite spatial networks, and for the simulations, are available in the Dryad repository; https://doi.org/10.5061/dryad.9w0vt4bjb<br>Implementation of the fixed-rows, 'fixed-columns' null model was performed using the r2dtable function from the 'stats' package (version 4.3.0) for R. Trajectory segmentation was carried out using the 'changepoint' package (version 2.2.2) for R.

For manuscripts utilizing custom algorithms or software that are central to the research but not yet described in published literature, software must be made available to editors and reviewers. We strongly encourage code deposition in a community repository (e.g. GitHub). See the Nature Portfolio guidelines for submitting code & software for further information.

Data

Policy information about availability of data

All manuscripts must include a data availability statement. This statement should provide the following information, where applicable:

- Accession codes, unique identifiers, or web links for publicly available datasets
- A description of any restrictions on data availability
- For clinical datasets or third party data, please ensure that the statement adheres to our policy

The automated tracking datasets are available in the Dryad repository, https://doi.org/10.5061/dryad.9w0vt4bjb

Human research participants

Policy information about studies involving human research participants and Sex and Gender in Research.

Reporting on sex and gender	N/A
Population characteristics	N/A
Recruitment	N/A
Ethics oversight	N/A

Note that full information on the approval of the study protocol must also be provided in the manuscript.

Field-specific reporting

Please select the one below that is the best fit for your research. If you are not sure, read the appropriate sections before making your selection.

☐ Life sciences ☐ Behavioural & social sciences ☒ Ecological, evolutionary & environmental sciences

For a reference copy of the document with all sections, see nature.com/documents/nr-reporting-summary-flat.pdf

Ecological, evolutionary & environmental sciences study design

All studies must disclose on these points even when the disclosure is negative.

Study description	Comparison of the spatial & social organization within nests of four species of social insects.
Research sample	The experiments were non-manipulative tracking experiments, conducted on 50 colonies of social insects.<br>As a purely descriptive study, there were no treatment factors, and the design was not factorial nested or hierarchical.<br>The 50 colonies were drawn from four species (Apis mellifera, 10 colonies; Lasius niger, 20 colonies; Leptothorax acervorum, 10 colonies; Temnothorax albipennis, 10 colonies).<br>For full details on the number of tracked individuals in each colony, see Table 1 in the main text.
Sampling strategy	There were two levels of replication within our study: the level of the colony and the level of the individual.<br>We chose to use 10 colonies per species (20 in the case of L. niger) as this represents a good practical compromise between sufficient replication at the colony level, and the time required to tag individuals that make up each colony.<br>In total, our resulting sample size was therefore of 50 colonies and 12494 individuals, totalling 340 000 ant/bee hours of observations.
Data collection	A. mellifera; 10x72hr tracking sessions by TOR; L. niger; 20x72hr sessions by NS; L. acervorum & T. nylanderi; 20x72hr sessions by TOR & NS.
Timing and spatial scale	ervorum & T. nylanderi; 20x72hr sessions by TOR & NS.<br>Timing: Tracking sessions were conducted betweeen November 2013 - December 2016.<br>Spatial scale - ants: Trajectories of individuals moving within ~60x40mm rectangular nests<br>Spatial scale - honeybees; Trajectories of individuals moving within ~60x40cm double-sided observation hives.
Data exclusions	No data were excluded.
Reproducibility	The experiments included internal replication (10 colonies per species). All reported findings were shown to be statistically significant using all replicates.
Randomization	Colonies were allocated to categories according to species.

Blinding	All data collection and analyses were performed using automated procedures (automated tracking system; custom computer software) and were therefore not subject to any human biases.

Did the study involve field work? ☐ Yes ☒ No

Reporting for specific materials, systems and methods

We require information from authors about some types of materials, experimental systems and methods used in many studies. Here, indicate whether each material, system or method listed is relevant to your study. If you are not sure if a list item applies to your research, read the appropriate section before selecting a response.

Materials & experimental systems

n/a	Involved in the study
☒	☐ Antibodies
☒	☐ Eukaryotic cell lines
☒	☐ Palaeontology and archaeology
☐	☒ Animals and other organisms
☒	☐ Clinical data
☒	☐ Dual use research of concern

Methods

n/a	Involved in the study
☒	☐ ChIP-seq
☒	☐ Flow cytometry
☒	☐ MRI-based neuroimaging

Animals and other research organisms

Policy information about studies involving animals; ARRIVE guidelines recommended for reporting animal research, and Sex and Gender in Research

Laboratory animals	The study involved colonies Apis mellifera, Lasius niger, Leptothorax acervorum, Temnothorax nylanderi.T
Wild animals	None
Reporting on sex	N/A
Field-collected samples	None
Ethics oversight	The use of insects in research is not subject to any regulations or ethical restrictions.

Note that full information on the approval of the study protocol must also be provided in the manuscript.
